# Revealing nanoscale sorption mechanisms of gases in a highly porous silica aerogel

**DOI:** 10.1107/S1600576724006794

**Published:** 2024-08-19

**Authors:** Phung Nhu Hao Vu, Andrzej P. Radlinski, Tomasz Blach, Ralf Schweins, Hartmut Lemmel, John Daniels, Klaus Regenauer-Lieb

**Affiliations:** a School of Materials Science and Engineering UNSW Sydney Sydney New South Wales2052 Australia; b WA School of Mines: Minerals, Energy and Chemical Engineering Curtin University Perth Western Australia6102 Australia; c Queensland Micro Nanotechnology Centre Griffith University Nathan Queensland4111 Australia; d DS/LSS Institut Laue–Langevin 71 avenue des Martyrs 38000Grenoble France; e Atominstitut TU Wien Stadionallee 2 1020Wien Austria; f Institut Laue–Langevin 38000Grenoble France; Australian Centre for Neutron Scattering, ANSTO, Australia

**Keywords:** methane adsorption, silica aerogels, contrast-matched small-angle neutron scattering, carbon dioxide sequestration, gas uptake

## Abstract

Geological formations provide a promising environment for the long-term and short-term storage of gases, including carbon dioxide, hydrogen and hydro­carbons, controlled by the rock-specific small-scale pore structure. This study investigates the nanoscale structure and gas uptake in a highly porous silica aerogel (a synthetic proxy for natural rocks) using transmission electron microscopy, X-ray diffraction, and small-angle and ultra-small-angle neutron scattering with a tracer of deuterated methane (CD_4_) at pressures up to 1000 bar. The results show that the adsorption of CD_4_ in the porous silica matrix is scale dependent. The pore space of the silica aerogel is fully accessible to the invading gas, which quickly equilibrates with the external pressure and shows no condensation on the sub-nanometre scale. In the 2.5–50 nm pore size region a classical two-phase adsorption behaviour is observed. The structure of the aerogel returns to its original state after the CD_4_ pressure has been released.

## Abstract 2

This research uses contrast-matched small-angle neutron scattering to investigate the adsorption behaviour of deuterated methane in a silica aerogel in the pressure range from 0 to 1000 bar. The results reveal a classical reversible two-phase adsorption in the 2.5–50 nm pore size region and no evidence of condensation in the sub-nanometre pores.

## 1. Introduction

The structure of nanoporous materials (such as porosity and pore size distribution) and their interaction with penetrating fluids (including permeability, pore accessibility and fluid/solid interactions at the interface) are crucial for various applications, including gas storage, separation and catalysis. Nano­structural details directly affect the sorption kinetics, sorption capacity and long-term storage stability and, as a result, the amount of energy required for the injection/production of fluids hosted in the pore space.

To gain insight into gas sorption phenomena in sedimentary rocks, we study here the nanoscale properties of a silica aerogel, a relatively simple engineered material. Silica and carbon aerogels are popular materials used in fundamental research (Melnichenko *et al.*, 2006 ▸; Chathoth *et al.*, 2010 ▸; Ciccariello *et al.*, 2011*a* ▸,*b* ▸) since they contain polydisperse micro- and nanopores, with sizes extending over several orders of magnitude, and have high porosity and a physically and chemically homogeneous solid matrix. In addition, the main components of the aerogels are also present in rocks (*e.g.* silica for sandstone and carbon for coal/shale).

Small-angle neutron scattering (SANS) has been widely adopted for microstructural studies of geological samples (Radlinski & Hinde, 2002 ▸; Radlinski *et al.*, 2004 ▸*b*; Clarkson *et al.*, 2013 ▸; Sun *et al.*, 2020 ▸; Radlinski *et al.*, 2021 ▸; Radlinski & Blach, 2023 ▸) due to its ability to access the pore space structure in a non-destructive manner and its capability of surveying a large range of pore sizes [ranging from sub-nanometre to tens of micrometres when combined with the ultra-small-angle neutron scattering (USANS) technique]. Importantly, SANS/USANS can also be employed independently to investigate the open (accessible) and closed (in­accessible) pore space by contrast matching the rock matrix with selected fluids (most frequently containing deuterium) such as pressurized deuterated methane (d-methane, CD_4_) or water/heavy water (H_2_O/D_2_O) mixtures (Bahadur *et al.*, 2018 ▸; Sun *et al.*, 2019 ▸; Sun *et al.*, 2020 ▸; Blach *et al.*, 2021*a* ▸; Radlinski *et al.*, 2021 ▸; Sun *et al.*, 2022 ▸).

During a contrast-matched (CM) SANS experiment using CD_4_, the pore–matrix contrast is reduced by increasing the external pressure supplied to the sample compartment, which affects (gradually reduces) the scattering intensity. It is often observed, however, that in the high-*Q* limit [usually for *Q* ≥ 0.1 Å^−1^, which corresponds to pore sizes smaller than 5 nm; *Q* = (4π/λ)sin(θ/2), where θ is the scattering angle and λ is the neutron wavelength] starting from pressures very much below the CM point, the SANS intensity increases rather than decreases as the gas is introduced into the system (Clarkson *et al.*, 2013 ▸; Ruppert *et al.*, 2013 ▸; Bahadur *et al.*, 2018 ▸; Jubb *et al.*, 2020 ▸; Blach *et al.*, 2021*a* ▸; Radlinski *et al.*, 2021 ▸; Jubb *et al.*, 2023 ▸). This indicates a different pore-filling mechanism for large and small pores, most likely due to rapid fluid condensation in tight confinement, thus forming a dense adsorbed (third) phase within the small nanopores and sub-nanopores. In addition, it has been observed for a number of rocks that despite the low accessibility of the larger pores (shown by only small differences between the intensity profiles under ambient and CM conditions) the SANS intensities in the sub-nanopore region differ much more significantly. This demonstrates that gas can efficiently migrate to and condense in the smaller nanopores despite having limited access to larger pores.

Formation of a third (dense adsorbed fluid) phase in confinement has not only been observed in small nanopores. An adsorption study of carbon dioxide in silica aerogels (Melnichenko *et al.*, 2006 ▸; Ciccariello *et al.*, 2011*a* ▸,*b* ▸), used as a proxy for more complex natural silicate rocks, showed that dense CM fluid was also present at the larger pore scale up to 50 nm. The authors suggested that the estimated density of the adsorbed fluid could be a weighted average of two distinct phases: a dense phase located close to the wall and a bulk-like gas phase, distributed depending on the applied gas pressure. For supercritical CO_2_, the density of the dense CO_2_ film could be as high as almost four times the density of the bulk CO_2_.

In this work, we investigate the interaction between the molecules of pressurized CD_4_ and the solid matrix of a silica aerogel over the pore size range (length scale, calculated as 2*r* = 5/*Q*) from 0.3 to 350 nm, accessible to the SANS experimental technique. This range is relevant to both the technology of CO_2_ geo-sequestration and the industrial-scale production of natural gas (methane). An engineered silica aerogel was chosen due to its chemical purity, high porosity and good accessibility of the pore space. In addition, using a silica-based rather than carbon-based aerogel inhibits possible complications caused by carbon–carbon fluid–matrix inter­actions, thus limiting the potential for chemical reactions and focusing on condensation effects caused by physical processes. We note that, unlike carbon dioxide, the interaction of methane with silica aerogels has not been previously studied; the current results can be used to compare the behaviour of the two different greenhouse gases in an SiO_2_-based porous matrix system.

## 2. Background of SANS

The scattering cross section (dΣ/dΩ)(*Q*) for cold neutrons [used interchangeably with the scattering intensity *I*(*Q*)] is governed by the value of the contrast 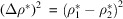, where 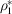 and 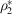 are the scattering length densities (SLDs) of each uniform phase (in this context, there are two phases: the invading fluid and the aerogel solid matrix). The magnitude of the SLD depends on the isotopic composition and density of a given phase (Melnichenko, 2015 ▸),
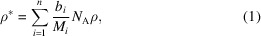
where *b_i_* is the coherent scattering length and *M_i_* is the atomic mass of every nucleus *i* in the molecule, *N*_A_ is Avogadro’s constant, and ρ is the bulk density.

The scattering intensity critically depends on the pore structure and geometry of the sample. For an isotropic two-phase system the structure is described by the correlation function γ(*r*) and *I*(*Q*) between the two phases of the chosen porous medium, given by the Debye–Porod formula (Debye *et al.*, 1957 ▸),
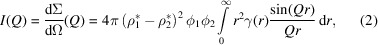
where ϕ_*i*_ (*i* = 1 or 2) is the total porosity of phase *i* (Melnichenko, 2015 ▸). This general formula can be used to calculate the scattering cross section using an independently determined correlation function, which has been done for numerous objects of both Euclidean and fractal geometry (Radlinski & Hinde, 2002 ▸; Radlinski *et al.*, 2004*a* ▸,*b* ▸; Melnichenko *et al.*, 2006 ▸; Chathoth *et al.*, 2010 ▸; Ciccariello *et al.*, 2011*a* ▸,*b* ▸; Clarkson *et al.*, 2013 ▸; Ruppert *et al.*, 2013 ▸; Bahadur *et al.*, 2018 ▸; Sun *et al.*, 2019 ▸; Sun *et al.*, 2020 ▸; Blach *et al.*, 2021*a* ▸; Radlinski *et al.*, 2021 ▸; Sun *et al.*, 2022 ▸). Conversely, the correlation function can be computed from the scattering intensity via the inverse Fourier transform.

For an isotropic system with a power-law (fractal-like) pore size distribution, the scattering intensity can be approximated by (Martin, 1986 ▸; Mildner & Hall, 1986 ▸)
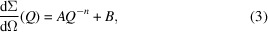
where *A* (the prefactor) and *B* (the background) are constants. Equation (3) represents the scattering intensity *I*(*Q*), composed of two components: the fractal-like scattering which follows the power law, and a flat background *B*. In the fractal *Q* range, the plot of *I*(*Q*) is linear on a log–log scale with a negative slope −*n*, where the value of *n* is related to the fractal dimension of the pore–matrix interface. The power-law exponent *n* can vary between 2 and 4; 2 < *n* < 3 indicates scattering from a mass fractal with dimension *D*_m_ = *n*, whereas 3 < *n* < 4 indicates scattering from a surface fractal with dimension *D*_s_ = 6 − *n*. A value of *n* = 3 corresponds to scattering from a very rough interface, whereas *n* = 4 is the result of smooth surface scattering (Wong & Bray, 1988 ▸).

For a two-phase system with a flat interface on the 1/*Q* scale, the scattering intensity is governed by Porod’s law (Porod, 1951 ▸):
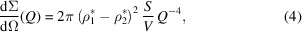
where SSA = *S*/*V* is the specific surface area of the scattering object. For a system with a smooth scattering interface, the plot of *Q*^4^*I*(*Q*) versus *Q*, called the Porod plot, shows a plateau (horizontal limit) in the corresponding *Q* range; the value of the SSA can then be estimated from the contrast between the two phases:
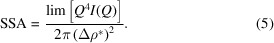
For a smooth non-flat interface, *i.e.* where there is a curvature at a scale corresponding to ∼1/*Q*, the deviation from the Porod law can be described using the Kirste–Porod formula (Kirste & Porod, 1962 ▸),
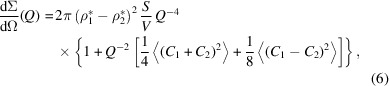
where *C*_1_ and *C*_2_ are the local principal curvatures of the surface, with the average 〈 〉 taken over the entire surface illuminated by neutrons.

For a specific system consisting of spherical particles of finite size, the scattering intensity for a single sphere in the Porod limit assumes the following form (Auvray & Auroy, 1991 ▸):
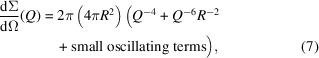
where the spherical radius of the system can be derived from the position of the first bump in the Porod plot as *R* ≃ 3/*Q*.

The Porod invariant *Q*_inv_ can be used to evaluate the total porosity for a two-phase system (with porosity ϕ_1_ of phase 1 and ϕ_2_ = 1 − ϕ_1_ of phase 2), regardless of the shape and geometry of the scattering objects (Porod, 1952 ▸):
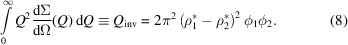
For reliable estimations of porosity, the computation of *Q*_inv_ requires scattering data to be collected over a wide enough *Q* range. Note that equation (8) is symmetric with respect to phase 1 and phase 2, meaning that the scattering intensity (and *Q*_inv_) will not change if each phase is replaced by its counterpart with the corresponding volume fraction (Babinet, 1837 ▸). For silica aerogels the void phase is dominant; the solid phase occupies only a few per cent of the volume. This information can be used to determine the correct volume fraction of the solid silica matrix.

For a system composed of polydisperse objects with a (roughly) spherical geometry, SANS results spanning a wide enough *Q* range (length scales) can be approximately described using the polydisperse spherical pore (PDSP) model (Hinde, 2004 ▸; Radlinski *et al.*, 2004*a* ▸),
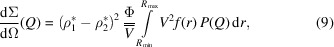
where 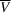 is the average pore volume, *f*(*r*) is the probability density function of the pore sizes and *P*(*Q*) is the spherical form factor. By fitting the PDSP model to experimental SANS data, the pore size distribution and the specific surface area can be obtained (Blach *et al.*, 2021*a* ▸,*b* ▸; Ji *et al.*, 2023 ▸).

For a polydisperse system of spherical objects which exhibit power-law scattering (interpreted as a proxy for a fractal-geometry medium), the relationship between the scattering vector and the pore size in real space is (Radliński *et al.*, 2000 ▸)
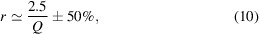
where *r* is the average pore size (radius) contributing the most to the scattering intensity measured at a scattering vector magnitude *Q*. For very rough surfaces (*D*_s_ = 2.9), scattering in the range *r* ≃ 2.5/*Q* ± 50% accounts for *ca* 66% of the total measured intensity; this contribution gradually decreases for smaller surface fractal dimensions (Radliński *et al.*, 2000 ▸). The remaining intensity at a given *Q* value originates from the rest of the pores within the entire *R* distribution.

## 3. Methodology

### 3.1. Sample preparation

The aerogel block (with side dimensions of the order of 1 cm) was originally obtained from Ocellus Technologies, Livermore, California, USA (purchased through BuyAerogel.com, https://www.buyaerogel.com/product/precision-silica-aerogel-square-tile/), and provided by Dr Yuri Melnichenko of Oak Ridge National Laboratory, Tennessee, USA. The exact procedure used to manufacture the sample is not known, but by analogy with other silica aerogel samples obtained from this source, its surface is probably covered by methoxy groups (Si—O—CH_3_) that form during the process of drying the precursor gel in supercritical methanol (Tajiri *et al.*, 1995 ▸; Soleimani Dorcheh & Abbasi, 2008 ▸; Ciccariello *et al.*, 2011*a* ▸). According to the manufacturer, the BET surface area of this aerogel is 600–1000 m^2^ g^−1^ with a bulk density of 0.09 g cm^−3^, a mean pore diameter of 20 nm and porosity of 96% (https://www.buyaerogel.com/product/precision-silica-aerogel-square-tile/).

The aerogel sample for CM SANS experiments was prepared by gently crushing fragments of the aerogel block into smaller chunks (of sub-millimetre sizes) and loosely depositing them inside a perforated cylindrical aluminium container with an internal thickness of 1 mm. The sample thickness used for the reduction of SANS data to absolute units of cm^−1^ was assumed also to be 1 mm. The effective sample thickness is smaller, owing to the incomplete filling of the sample container volume. A correction factor of 0.68 (= *d*_app_/*d*_aerogel_, where the apparent sample density is *d*_app_ = *m*_sample_/*V*_container_, with *m*_sample_ = 0.007565 g and *V*_container_ = 0.123 cm^3^) was then used to calibrate the measured scattering intensity for the effective thickness.

### 3.2. CM SANS sample environment

The sample, encapsulated in a cylindrical aluminium holder with an internal diameter of 12.5 mm and internal thickness of 1 mm, was mounted inside a custom-built SANS pressure cell (Ji, 2020 ▸; Ji *et al.*, 2024 ▸), which is an improved version of the ORNL-2 cell (Melnichenko, 2015 ▸). Prior to the measurements, the aluminium sample holder and the sample compartment inside the pressure cell were cleaned using alcohol, acetone and di­chloro­methane.

For CM SANS experiments, a controlled volume of pressurized CD_4_ was introduced into the sample compartment (the space between two internal titanium windows, separated by a distance corresponding to the external size of the aluminium sample holder), enabling measurements at a number of pressure steps ranging from vacuum to 1000 bar. The free volume available to the pressurized gas and exposed to the neutron beam was confined inside the sample container. The pressure stability during the measurements was of the order of 5 bar.

Experiments were performed at the uncontrolled temperature of the experimental hall of 22°C at the following pressure steps: vacuum – 100 bar – 250 bar – 500 bar – 600 bar – 700 bar – 800 bar – 900 bar – 1000 bar – 450 bar – 400 bar – 350 bar – 300 bar – 200 bar – 150 bar – 50 bar – vacuum. Since the scattering length density of CD_4_ is SLD(CD_4_; *P*, *T*) = 1.0 × 10^11^ × *d*(CD_4_; *P*, *T*) (in cm^−2^), where *d* is the density of CD_4_ (in g cm^−3^) at pressure *P* and temperature *T*, the pressure range from vacuum to 1000 bar corresponds to SLD values ranging from 0 to 4.2 × 10^10^ cm^−2^. For calculations it was assumed that *d*(CD_4_; *P*, *T*) = 1.25 × *d*(CH_4_; *P*, *T*). The pressure dependence of the density of methane on bulk pressure at a temperature of 22°C, *d*(CH_4_; *P*, 22°C), was obtained using the NIST Chemistry WebBook (2017 ▸).

### 3.3. SANS/USANS measurements

SANS and USANS results for the silica aerogel at each pressure step were acquired using instruments D11 and S18 at the Institut Laue–Langevin, France (Lindner *et al.*, 1992 ▸; Kroupa *et al.*, 2000 ▸; Lindner & Schweins, 2010 ▸). The SANS measurements, performed at three sample-to-detector distances of 1.4, 8 and 39 m at a wavelength of 5 Å, covered a *Q* range from 1.5 × 10^−3^ to 0.5 Å^−1^. The total acquisition time at each pressure was about 40 min and the pressure equilibration time between the pressure steps was about 5 min. USANS data were collected using a wavelength of 1.92 Å, covering a *Q* range between 3 × 10^−5^ and 2 × 10^−4^ Å^−1^; the acquisition time for each pressure step was 6 h. Raw SANS and USANS data were reduced following standard procedures (Melnichenko, 2015 ▸). First, the instrument background and scattering of the empty pressure cell measured in vacuum (with the aluminium container in place) were subtracted. The scattering of a 1 mm thick H_2_O sample (a secondary calibration standard cross-calibrated against H/D polymer blends), with a known differential scattering cross section of 0.929 cm^−1^ at λ = 5 Å, was then used to convert the SANS intensity of the silica aerogel into absolute units of cm^−1^. The SANS and USANS data, however, could not be merged following the usual practice shown in previous (U)SANS studies (Clarkson *et al.*, 2013 ▸; Bahadur *et al.*, 2018 ▸; Radlinski & Mastalerz, 2018 ▸; Blach *et al.*, 2021*a* ▸; Radlinski *et al.*, 2021 ▸), since the limited *Q* range of USANS, caused by the weak scattering signal, had no overlap with the *Q* range of the SANS measurements.

In order to determine the contribution of pressurized CD_4_ to the scattering profile of the silica aerogel sample, additional control SANS measurements of the empty pressure cell at a CD_4_ pressure of 500 bar (with and without the aluminium sample holder in place) were performed using the Quokka instrument at the Australian Nuclear Science and Technology Organisation (Wood *et al.*, 2018 ▸).

### 3.4. Sample preparation for TEM and electron/X-ray diffraction

Transmission electron microscopy (TEM) was employed to provide direct visualization of the silica aerogel microstructure, while electron and X-ray diffraction were used to gain additional insights into the aerogel structure at the molecular scale. Samples were prepared for analysis using sonification, which involves adding small chunks of the aerogel to ethanol and creating a suspension by ultrasonic stirring. The suspension (20 µl) was deposited onto a 3 mm diameter Cu grid coated with a thin film of carbon. After the ethanol had evaporated, the particles of aerogel remained attached to the carbon surface. TEM data were acquired using the JEOL JEM-F200 multi-purpose microscope at the Mark Wainwright Analytical Centre at the University of New South Wales; a cold field-emission gun scanning transmission electron microscope operating at 200 kV in the transmission mode provided a structural resolution of 0.1 nm. Electron diffraction data were collected using the same instrument with a wavelength of 2.5 pm at an acceleration voltage of 200 keV, whereas X-ray diffraction data were collected using a wavelength of 1.54 Å. TEM images and electron diffraction data of the sample prepared by manual crushing were also acquired to ensure structural information on the sample was preserved during the sonification process.

## 4. Results and discussion

### 4.1. Nanostructure of the silica aerogel

#### 4.1.1. Electron/X-ray diffraction

**Figure 1:**
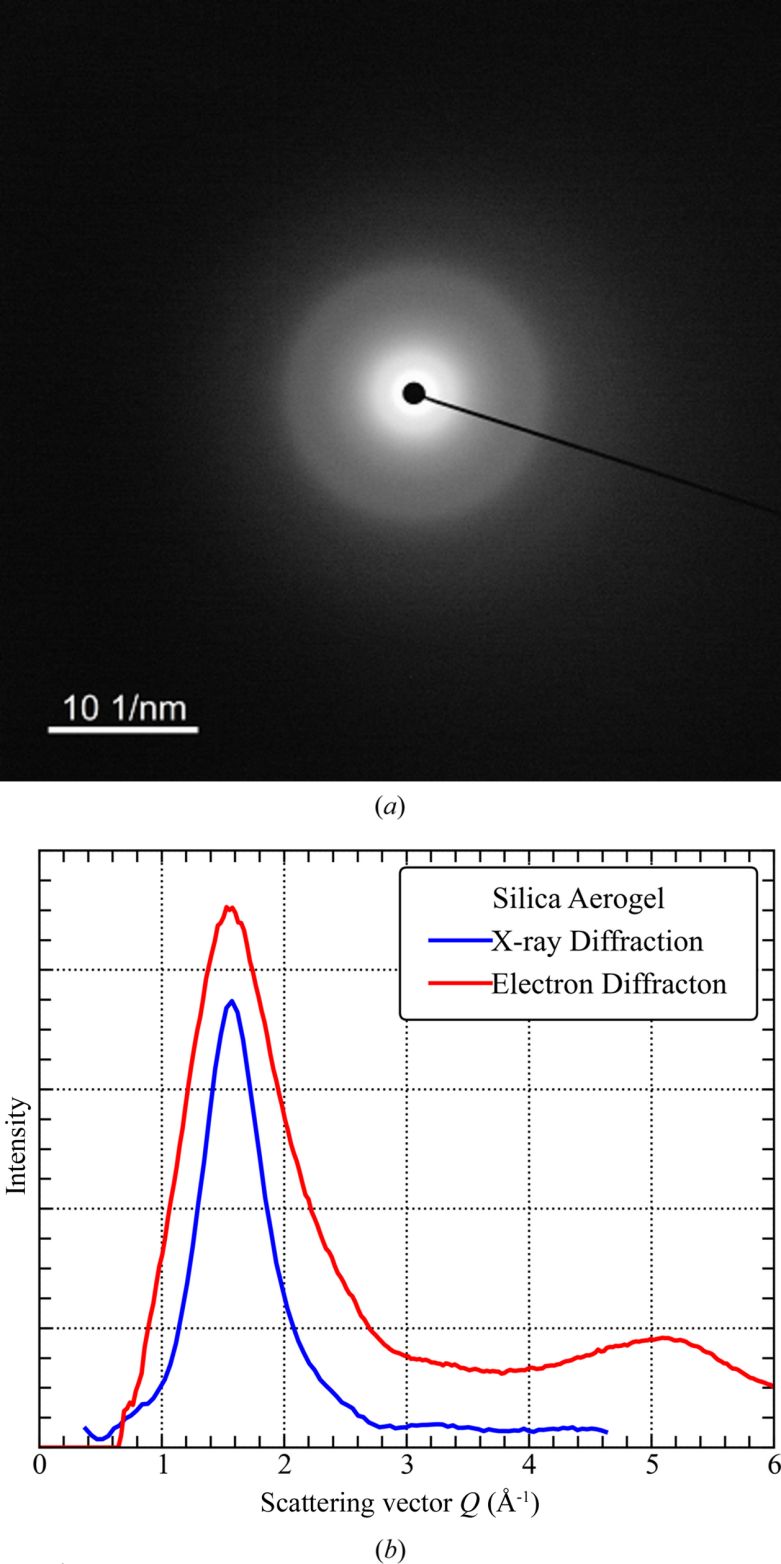
Diffraction data for the silica aerogel. (*a*) 2D electron diffraction spectrum acquired using an accelerating voltage of 200 keV. (*b*) Azimuthally averaged electron and X-ray diffraction intensity versus *Q*. Electron diffraction data were acquired in vacuum conditions and the XRD data under ambient conditions.

The silica aerogel is mostly amorphous (Fig. 1 ▸), with a primary peak centred at *Q* = 1.56 Å^−1^ (present in both the X-ray and the electron diffraction data); a smaller bump at *Q* = 5.15 Å^−1^ is seen in the electron diffraction data only. The two corresponding feature sizes, calculated using Bragg’s law (*r* = π/*Q*), are 0.2 and 0.06 nm, respectively. The peaks are most likely the result of the combination of Si—Si, Si—O and O—O bonds. However, it is not possible to resolve the details of these structures due to the amorphous nature of the silica aerogel.

The diffraction data pertain to the *Q* range characteristic of the interatomic distances, in contrast to SANS data which are characteristic of the larger, above-molecular scale; therefore there is no overlap between the two *Q* ranges.

#### 4.1.2. TEM imaging

**Figure 2:**
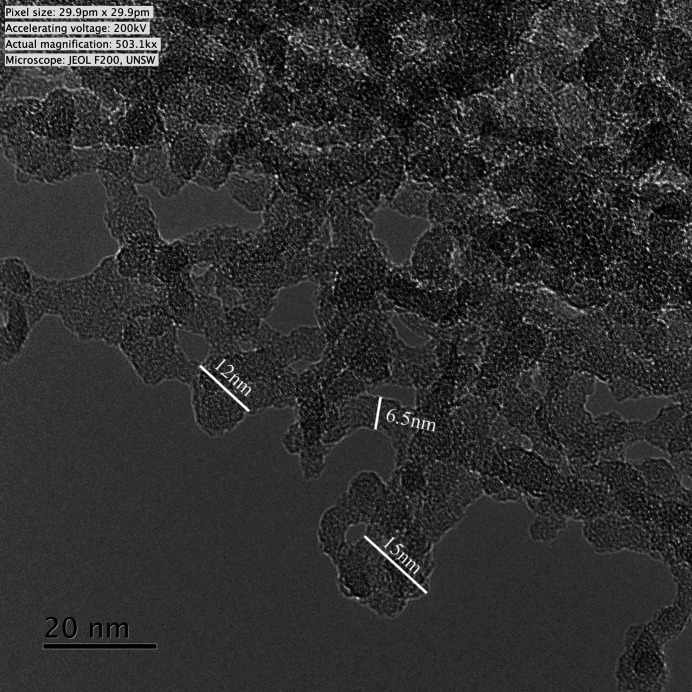
TEM image of the silica aerogel, taken under a magnification of 503 000× at an accelerating voltage of 200 kV.

The TEM image in Fig. 2 ▸ shows that the aerogel nanostructure consists of loosely connected clusters of amorphous silica with a diameter of about 6 nm each. The smallest clusters have diameters of the order of 10–20 nm and appear to be connected by ‘chain’ structures of 5–10 nm in diameter. The TEM image of the silica aerogel is consistent with the previously reported structure of mass fractals (Schaefer & Keefer, 1986 ▸; Foret *et al.*, 1992 ▸).

#### 4.1.3. SANS results at *P* = 0 (vacuum condition)

##### 4.1.3.1. General form of SANS intensity

**Figure 3:**
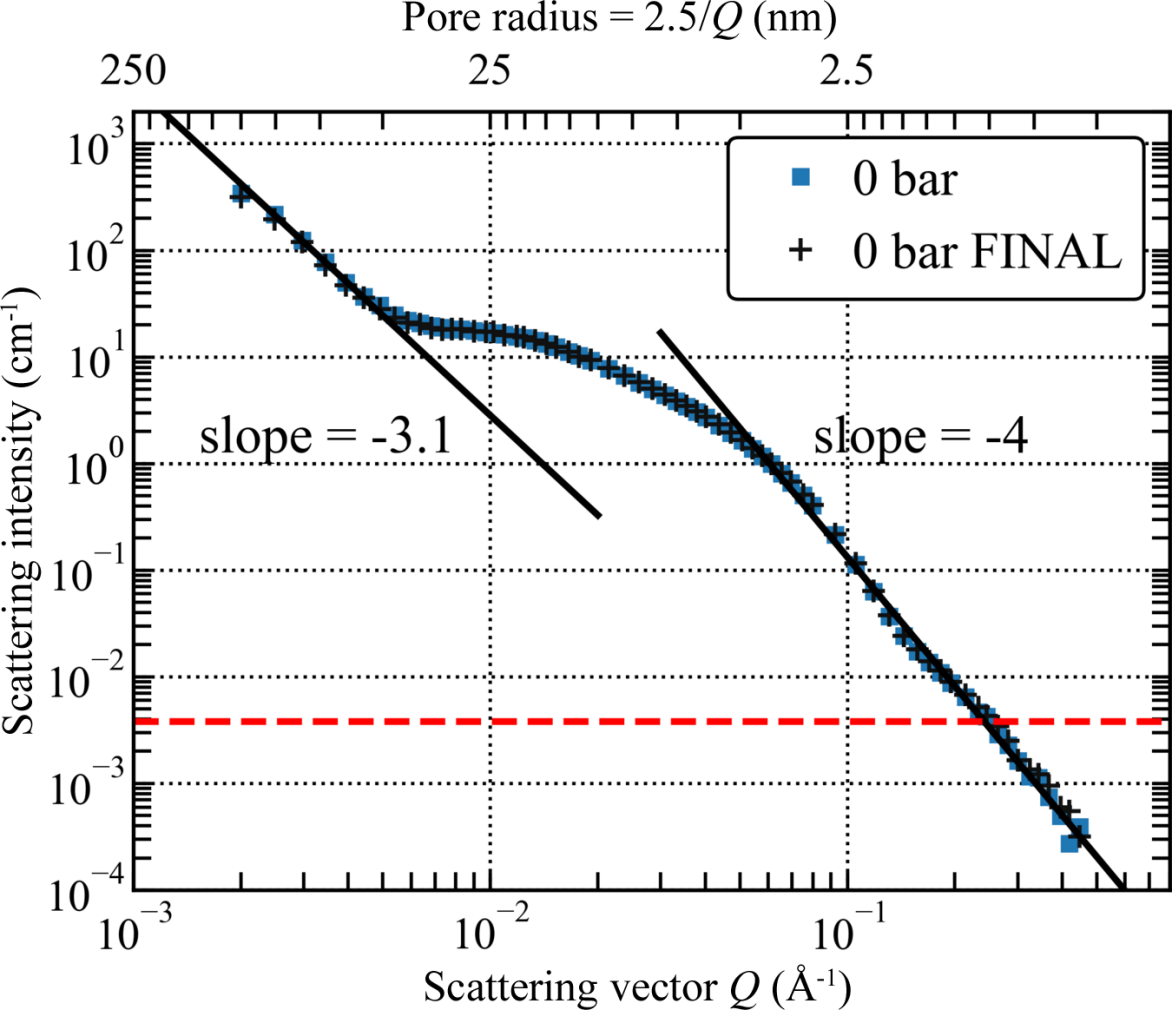
Background-subtracted SANS intensity for the silica aerogel measured in vacuum before (blue squares) and after (black crosses) pressure cycling with CD_4_ up to *P* = 1000 bar. The horizontal dashed line represents the subtracted flat background of 3.8 × 10^−3^ cm^−1^.

The *Q* dependence of SANS intensity, presented in Fig. 3 ▸, originates from a complex polydisperse system and displays three distinctive scattering regions: (i) for *Q* < 5 × 10^−3^ Å^−1^: low-*Q* region with a slope of about −3.1; (ii) for 5 × 10^−3^ < *Q* < 5 × 10^−2^ Å^−1^: mid-*Q* region with a broad scattering band; and (iii) at *Q* > 6 × 10^−2^ Å^−1^: Porod-like scattering from a smooth surface with a slope of −4.

**Figure 4:**
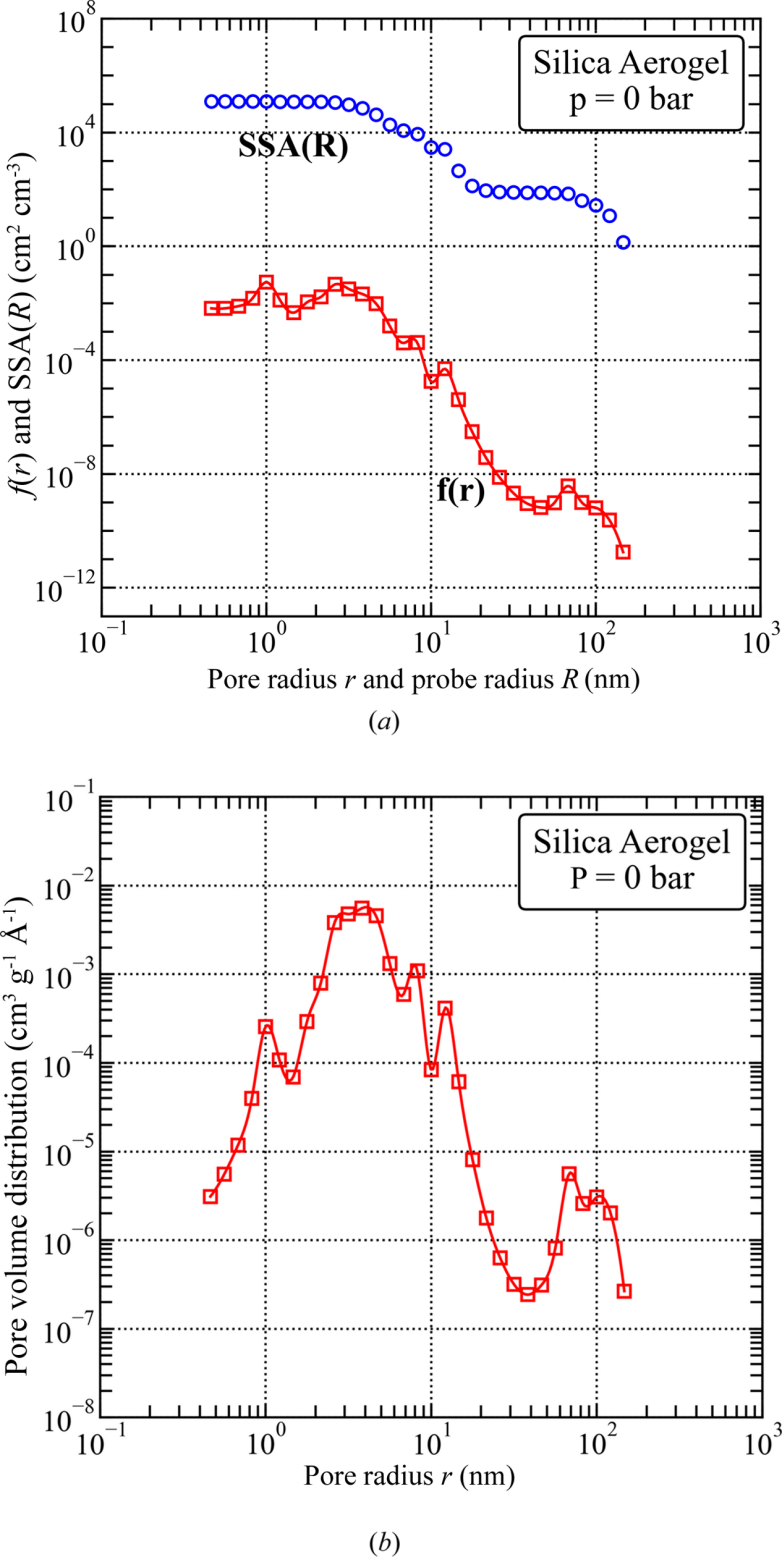
(*a*) Specific surface area plotted against probe size [SSA(*R*) versus *R*] and pore size distribution [*f*(*r*)] and (*b*) pore volume distribution for the silica aerogel in vacuum, obtained from fits of the PDSP model to SANS data. The SSA for a probe with a radius *R* is defined as the sum of SSAs of all pores with radii larger than *R*, divided by the sample volume.

A fit of the PDSP model to the SANS data provides a pore volume distribution with a prominent broad peak at a pore radius (used interchangeably with ‘pore size’ in the following) of 3.5 nm and two much smaller peaks at ∼1 and ∼80 nm (Fig. 4 ▸). Note that the position of the prominent peak is much smaller than the manufacturer-specified mean pore radius of 20 nm.

The fitted SSA is 1.4 × 10^5^ ± 2.5 × 10^3^ cm^2^ cm^−3^ for pores smaller than 3 nm in radius; the contrast value used for the void–matrix system is 3.2 × 10^10^ cm^−2^. The rough surface fractal-like scattering in the low-*Q* region (Fig. 3 ▸) and the high concentration of pores with radii close to 3.5 nm (diameter of 6 nm, Fig. 4 ▸) are consistent with the image provided by TEM (Fig. 2 ▸).

Upon exposure to pressurized CD_4_ (Section 4.2), the contributions to *I*(*Q*) from various pore sizes (different regions in *Q* space) vary due to the pore-size-dependent adsorption mechanisms. Importantly, however, the nano­structure of the aerogel remains unaffected by exposure to CD_4_ at pressures up to 1000 bar (Fig. 3 ▸).

##### 4.1.3.2. Volume fraction of solid silica estimated from Porod invariant

The lower limit of ϕ_1_ϕ_2_ = ϕ_1_(1 − ϕ_1_) and therefore the lower limit of the total porosity can be estimated from the Porod invariant *Q*_inv_ [equation (8)]. In vacuum, the contrast value for the silica aerogel matrix/void system is 3.2 × 10^10^ cm^−2^ and *Q*_inv_ is estimated at 5.15 × 10^20^ cm^4^ from the SANS data presented in Fig. 3 ▸. Since the experimental data do not fully extend to *Q* = 0, the invariant integral is underestimated, as is the calculated product ϕ(1 − ϕ) = 0.026. As a result, the value of ϕ is no less than, but close to, 2.6% for the solid fraction (97.4% pore), which is consistent with the porosity of 96% stated by the manufacturer (https://www.buyaerogel.com/product/precision-silica-aerogel-square-tile/).

##### 4.1.3.3. Nanoscale SSA estimated from Porod plot

**Figure 5:**
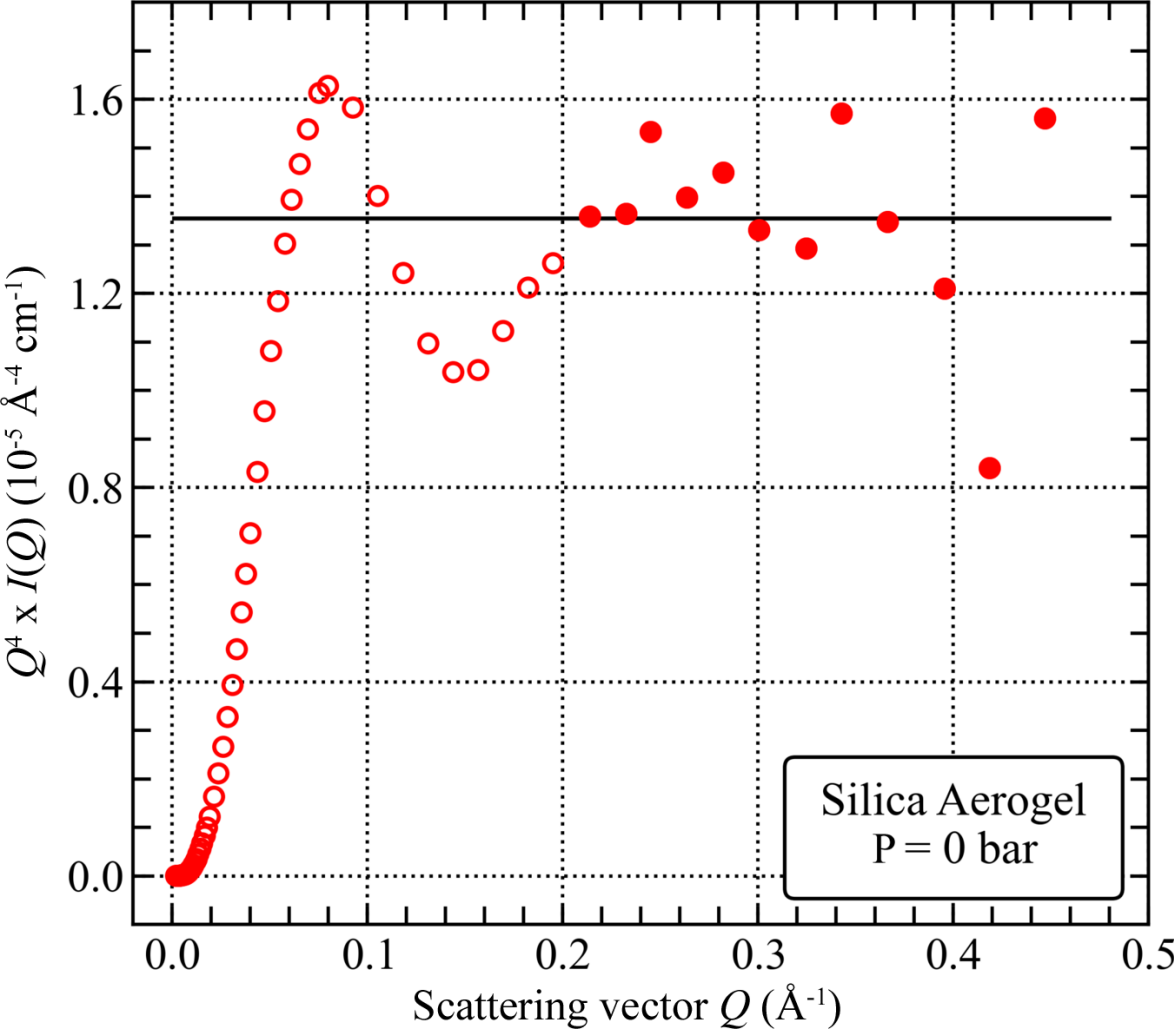
Porod plot of the silica aerogel in vacuum. Solid markers represent the data points being averaged to obtain the Porod limit (horizontal solid line).

The Porod plot (Fig. 5 ▸; prepared using SANS data after subtraction of the 3.8 × 10^−3^ cm^−1^ high-*Q* scattering background) of the silica aerogel in vacuum does not converge to a definitive Porod limit [equation (5)]. The significant scatter of the *Q*^4^*I*(*Q*) values in the high-*Q* limit is most likely due to the weak SANS signal in this *Q* range, further accentuated after subtraction of the high-*Q* background. A peak centred at *Q* = 0.082 Å^−1^ probably originates from the curvature of the pore–matrix interface on the nanoscale [equation (7)], with an estimated radius of curvature *R* ≃ 3/*Q* ≃ 3.8 nm, consistent with the position of the broad peak computed using the PDSP model [Fig. 4 ▸(*b*)]. Using (i) the approximate value of lim_*Q*→∞_[*Q*^4^*I*(*Q*)] equal to 1.35 × 10^−5^ Å^−4^ cm^−1^ (averaged from SANS data for *Q* > 0.2 Å^−1^) and (ii) the contrast value of 3.2 × 10^10^ cm^−2^ for the void–matrix system, the SSA value of 2.1 × 10^5^ ± 2.9 × 10^4^ cm^2^ cm^−3^ is estimated for scales smaller than ∼1.5 nm.

### 4.2. Pressure dependence of SANS results

#### 4.2.1. Salient features

**Figure 6:**
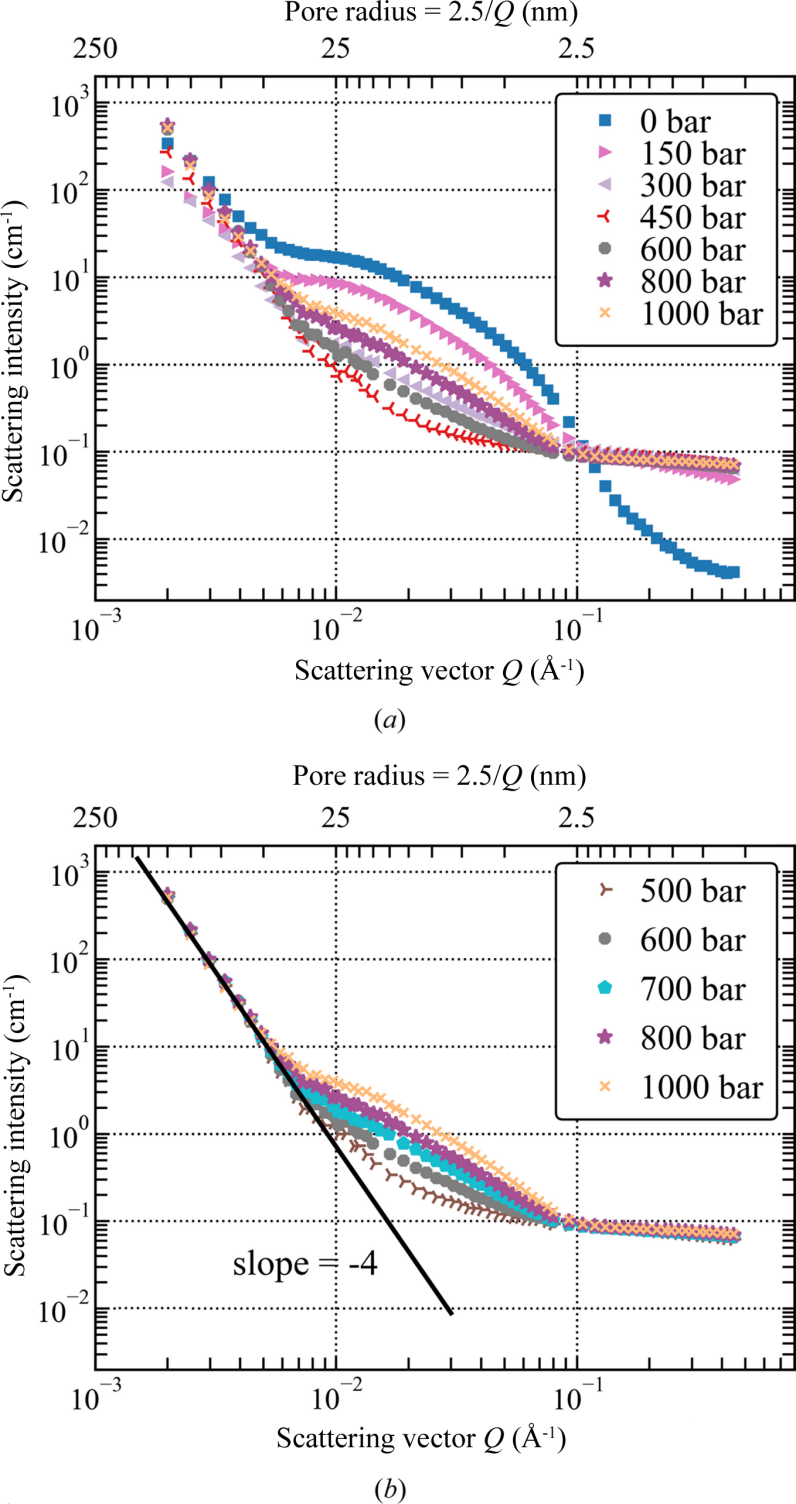
Variation in the absolutely calibrated SANS cross section for the silica aerogel with the pressure of CD_4_. (*a*) Data selected to illustrate the general trend in the pressure range from vacuum to 1000 bar and (*b*) results for all pressures higher than 500 bar.

**Figure 7:**
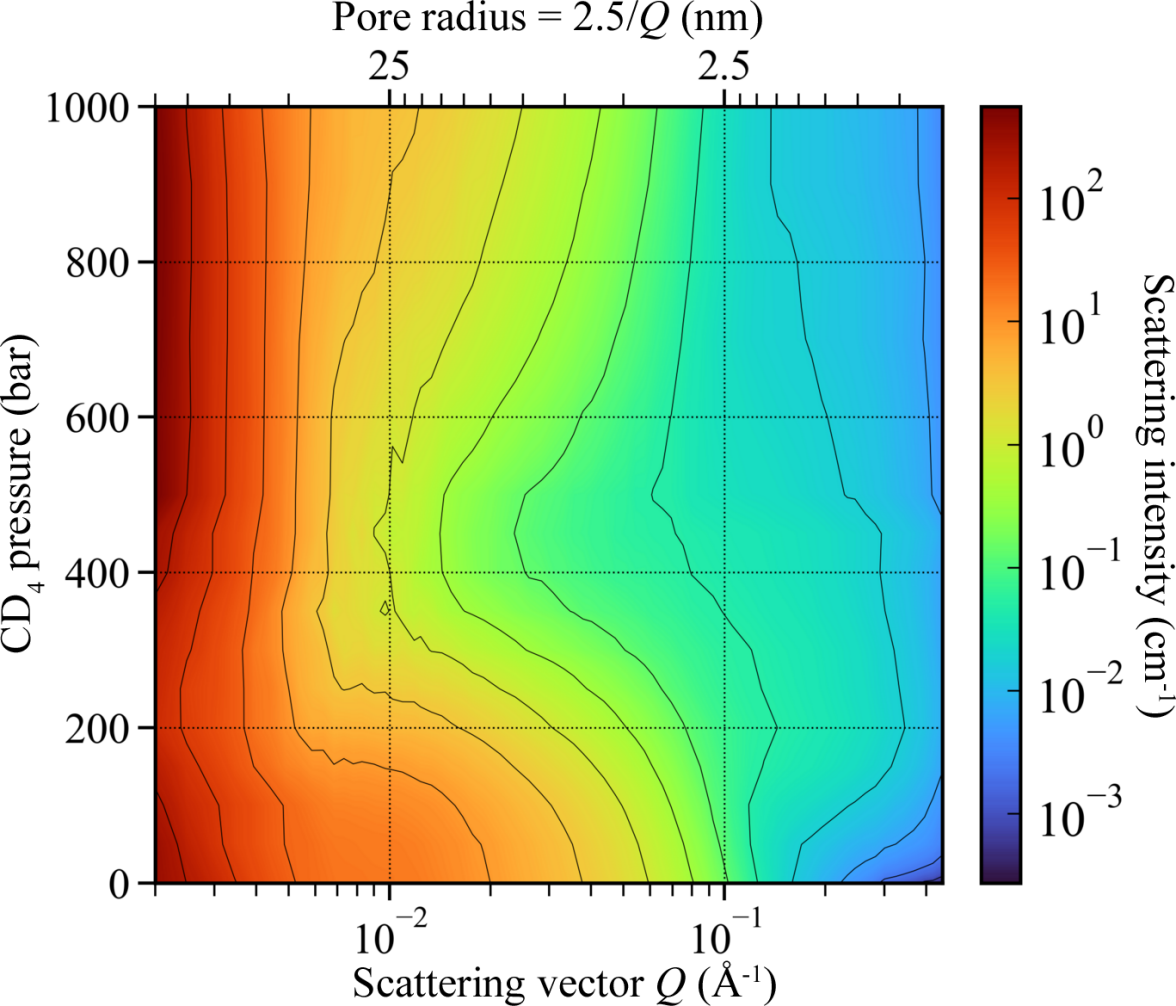
2D plot of the evolution of the scattering intensity profile *I*(*Q*) in the full *Q* range used in the SANS measurements as a function of the external (bulk) CD_4_ pressure.

The evolution of the azimuthally averaged SANS intensity with the pressure of CD_4_ is shown in Fig. 6 ▸ [as a series of *I*(*Q*) plots] and Fig. 7 ▸ (as a colour map). For clarity, only selected data acquired in the pressure range from vacuum to 1000 bar are shown in Fig. 6 ▸(*a*) and all SANS data acquired for pressures ≥500 bar are reproduced in Fig. 6 ▸(*b*).

The stepwise increase of CD_4_ pressure in the pore space of the silica aerogel causes a systematic change in the SANS intensity over the entire *Q* range. The following salient features are observed:

(i) In the high-*Q* range (*Q* > 0.2 Å^−1^, *r* < 1.3 nm), the scattering intensity increases by a factor of ∼10 between vacuum and *P* = 100 bar, then by a factor of ∼2 between 100 bar and 200 bar, and remains almost constant as the pressure gradually increases up to 1000 bar [Fig. 6 ▸(*a*)].

(ii) In the mid-*Q* range (0.01 < *Q* < 0.1 Å^−1^, 4 < *r* < 25 nm), the scattering intensity decreases to a minimum at *P* ≃ 450 bar and systematically increases with increasing pressure up to 1000 bar (Fig. 6 ▸).

(iii) In the low-*Q* range (*Q* < 5 × 10^−3^ Å^−1^, *r* > 80 nm), the SANS intensity initially decreases with pressure (with a minimum intensity at *P* = 200 bar, at about 31% of the intensity at *P* = 0); then in the range 250–500 bar the intensity increases (to about 1.6 times above the *P* = 0 level), and it stays at this plateau for pressures between 500 and 1000 bar (Figs. 6 ▸ and 8 ▸).

(iv) After the pressure of CD_4_ is released and the sample is re-exposed to vacuum, the SANS curve returns to its original shape and intensity (Fig. 3 ▸).

#### 4.2.2. Scattering background from pressurized CD_4_

The scattering of the empty cell in vacuum, *I*_MC_(*Q*; *P* = 0), is routinely subtracted from the SANS results as part of the data processing procedure, including in this work. Following the results of early SANS test measurements of the empty cell (Clarkson *et al.*, 2013 ▸; Bahadur *et al.*, 2018 ▸; Blach *et al.*, 2021*a* ▸; Radlinski *et al.*, 2021 ▸) it has usually been assumed that *I*_MC_ is only weakly affected by scattering of the pressurized CD_4_ compared with the scattering cross section of geological mater­ials, and hence *I*_MC_(*Q*; *P* > 0) ≃ *I*_MC_(*Q*; *P* = 0). This approximation significantly reduces (halves) the demand for experimental beam time. The absolute scattering cross section of silica aerogels [Fig. 7.9 of Melnichenko (2015 ▸)] in the SANS *Q* range is, however, one to two orders of magnitude smaller than that for shale (Radlinski *et al.*, 2021 ▸; Sun *et al.*, 2022 ▸) or coal (Zhang *et al.*, 2015 ▸; Radlinski & Mastalerz, 2018 ▸), and therefore the pressure dependence of *I*_MC_(*Q*; *P*) cannot be *a priori* ignored. Control measurements performed at *P* = 500 bar (Figs. S1 and S2 in the supporting information) reveal that *I*_MC_(*Q*; *P*) may be comparable to *I*_Si_(*Q*; *P*), especially at pressures close to the contrast match point; hence *I*_MC_(*Q*; *P*) may provide a significant contribution to the background scattering that is not accounted for during the standard data processing procedure, where *I*_MC_(*Q*; *P* = 0) is used.

In the absence of the complete set of *I*_MC_(*Q*; *P*) results, the *Q* dependence of the scattering background which originates from the pressurized CD_4_ and its interactions with the sample compartment components traversed by the neutron beam has been approximated by the sum of two (pressure-dependent) power-law functions. The procedure is discussed in detail in Appendix *C* in the supporting information.

#### 4.2.3. Adsorption of CD_4_ in nanopores

In the pressure interval from vacuum to 150 bar, the scattering intensity in the high-*Q* limit (at *Q* ≃ 0.5 Å^−1^, corresponding to a pore size 2.5/*Q* ≃ 0.5 nm) increases twentyfold from ∼3 × 10^−3^ to ∼0.06 cm^−1^ and then remains relatively stable up to *P* = 1000 bar (at a level of ∼0.1 cm^−1^). From the contrast considerations presented in Fig. S6 of Appendix *D* in the supporting information, such an increase is much too large to be consistent with CD_4_ condensation in the nanopores, a phenomenon widely observed in sedimentary rocks (Bahadur *et al.*, 2018 ▸; Radlinski *et al.*, 2021 ▸; Jubb *et al.*, 2023 ▸). In the high-*Q* region (from 0.1 to 0.5 Å^−1^, pore size range 0.5–2.5 nm) the SANS intensity tends to plateau at high pressures rather than follow the V-shaped pressure dependence expected for a two-phase system subjected to contrast matching. Importantly, the SANS patterns acquired at *P* = 500 bar for the pure CD_4_ fluid and the CD_4_-invaded silica aerogel sample [processed using the empty cell background *I*_MC_(*Q*; *P* = 0)] are similar: parallel on the log–log plot with a power exponent (slope) of −0.24 (Fig. S2). Unexpectedly, at the large-*Q* limits, the SANS profiles of pure CD_4_ measured with and without the aluminium container differ significantly from the SANS intensity of the pressurized aerogel with a scaling factor of 2.4 and 0.82, respectively.

Following these observations, we postulate that the variation in SANS intensity with pressure in the high-*Q* range (Fig. 6 ▸) has a large component that originates from nanoscale heterogeneities of the scattering contrast inside the sample compartment, which are not related to the presence of the sample. There is no evidence of CD_4_ condensation in the silica aerogel on the 0.5–2.5 nm scale, but it could be masked by the scattering from other objects; this is discussed in Appendix *B* in the supporting information.

#### 4.2.4. Adsorption of CD_4_ in 50–125 nm pores

**Figure 8:**
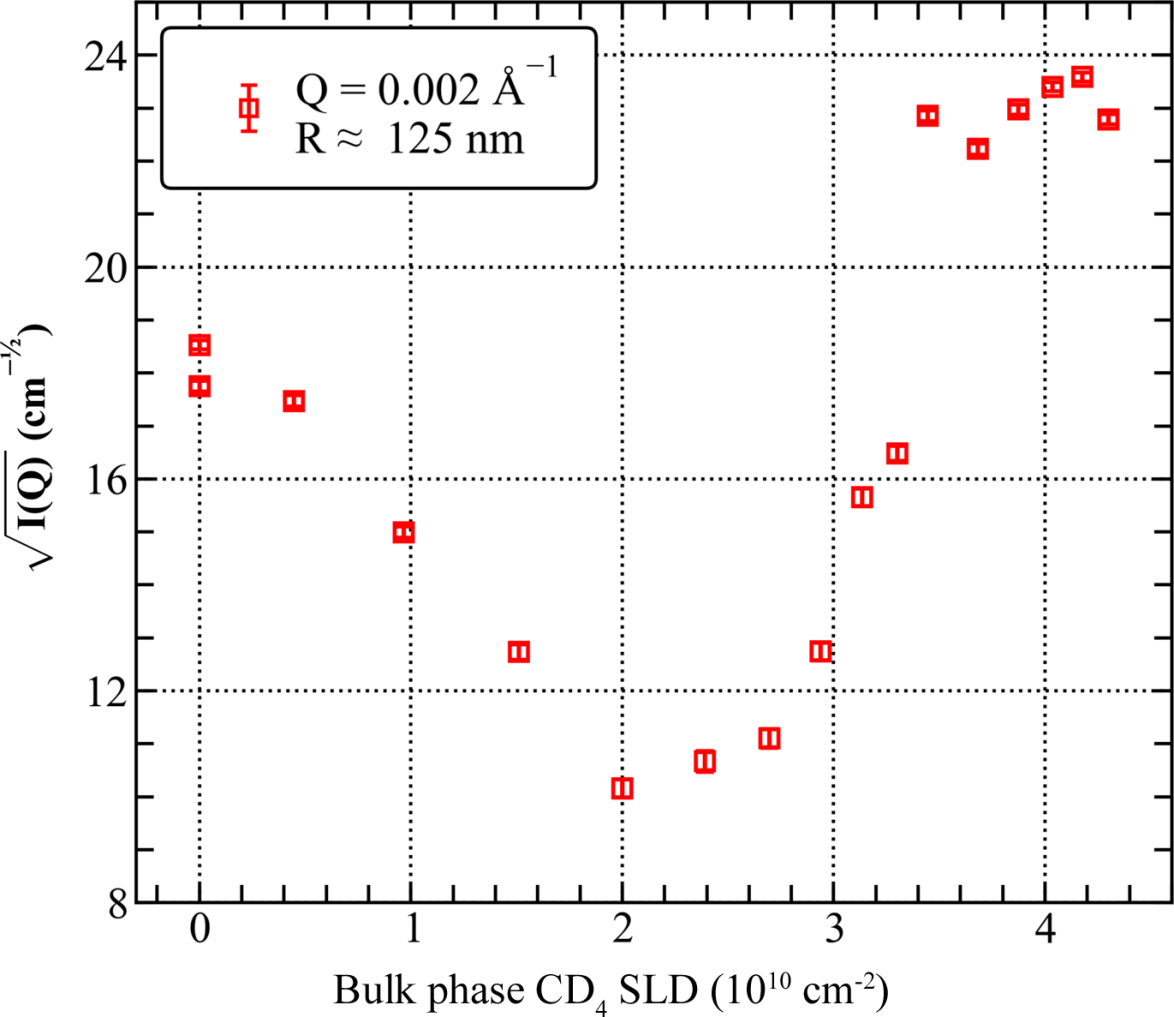
Square root of the SANS intensity measured at *Q* = 0.002 Å^−1^ in pressure steps from 0 to 1000 bar, presented as a function of the SLD of pressurized CD_4_.

The evolution of SANS intensity with pressure observed on the 2.5/*Q* scale of 50–125 nm [shown in Fig. 8 ▸ for a pore diameter of 125 nm, *i.e.* for *I*(*Q* = 2 × 10^−3^ Å^−1^; *P*)] suggests a mixed adsorption mechanism which involves more than one type of the CD_4_/solid matrix interface. The initial decrease in intensity is consistent with the onset of contrast matching with the aerogel matrix, but the minimum at SLD = 2 × 10^10^ cm^−2^ (*P* = 200 bar) corresponds to the interface with Al rather than SiO_2_. In addition, the broad minimum does not reach zero scattering intensity and extends to SLD = 3 × 10^10^ cm^−2^ (*P* = 300 bar), which indicates that interfaces with TiO_2_ and Ti may also contribute to the scattering; the latter since only the CD_4_/Ti contrast is large enough to explain why *I*(*Q*; *P* = 1000 bar) is 1.6 times larger than *I*(*Q*; *P* = 0). Furthermore, at CD_4_ pressures greater than or equal to 500 bar, the SANS data exhibit the classical *Q*^−4^ Porod behaviour, indicating scattering at a smooth interface.

**Figure 9:**
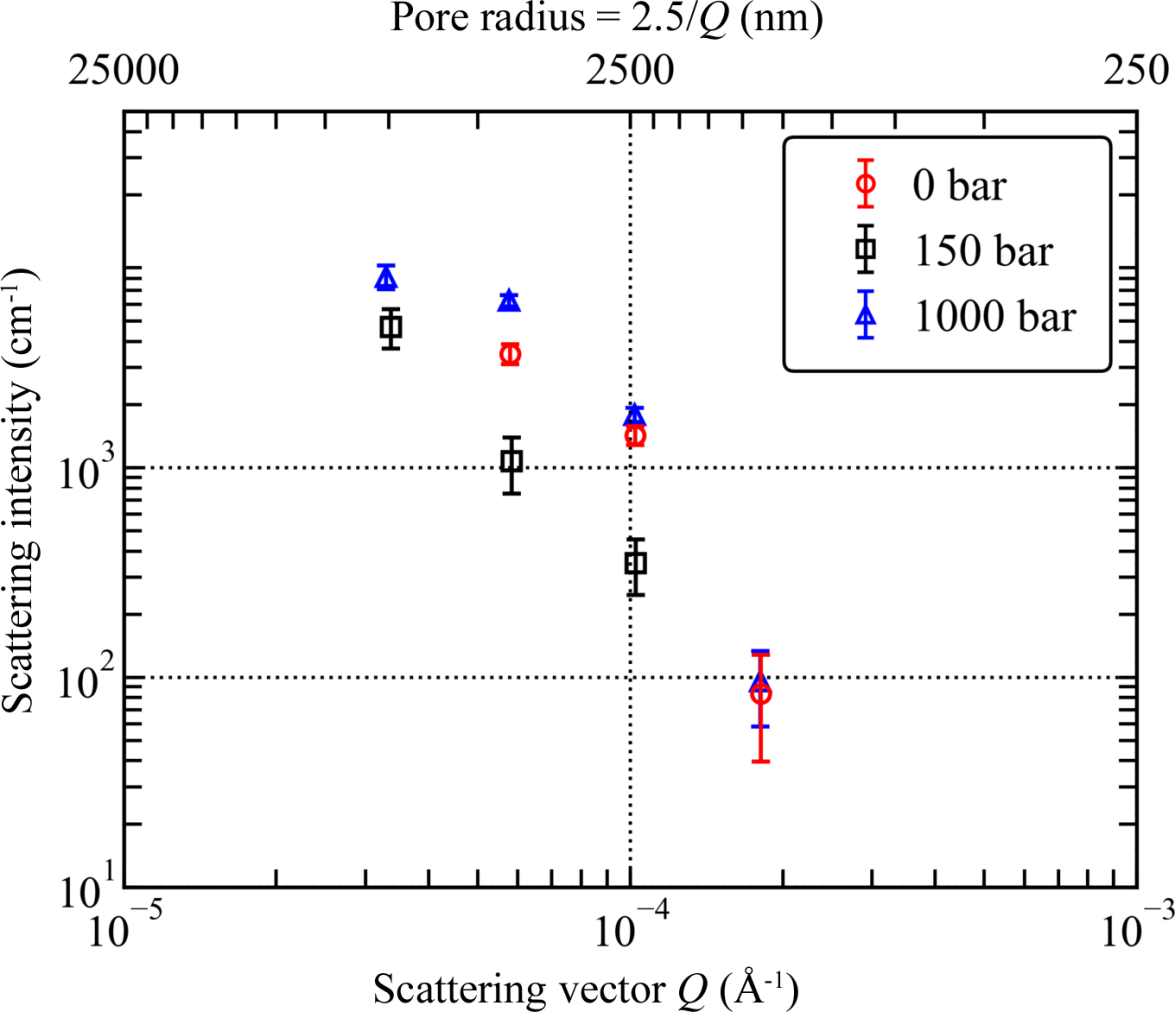
Variation in USANS data of the silica aerogel under vacuum and under CD_4_ pressures of 150 and 1000 bar.

USANS intensities measured in the *Q* range corresponding to micrometre-sized pores (Fig. 9 ▸) are qualitatively consistent with this interpretation. Due to the weak scattering signal, reliable data were acquired in a very limited *Q* range at three pressures of CD_4_. Significantly, the USANS intensity decreases as the CD_4_ pressure increases from vacuum to 150 bar, as expected in the two-phase approximation; for the CD_4_ pressure of 1000 bar, however, the USANS intensity exceeds the values measured in vacuum. The above contrast considerations are based on the SLD values listed in Table S3 and shown in Fig. S6 in the supporting information.

The evolution of the SANS slope with pressure in the low-*Q* region confirms a gradual transition from the rough-surface-fractal-like scattering characteristic of a silica aerogel in vacuum (slope = −3.1) to scattering at a flat surface for *P* ≥ 500 bar where the Porod limit is reached (Fig. 11). It is possible that the smooth surface scattering is caused by (i) growth of the adsorbed layer of CD_4_ on the surface of the silica matrix, possibly facilitated by the presence of methoxy groups (Si—O—CH_3_, a by-product of the Si aerogel production process), and/or (ii) the interface between the adsorbed molecules of CD_4_ and the (possibly oxidized) metal surfaces exposed to gas inside the sample compartment. The SSA of the latter can be roughly estimated from the area of the metal surfaces exposed to the neutron beam, which are (i) the internal surfaces of the titanium windows (two surfaces) and (ii) the outer and inner surfaces of the aluminium sample holder (four surfaces). The diameter of the neutron beam is 12.5 mm, and hence the illuminated surface area is 1.23 cm^2^; the estimated geometric surface area (in the scattering plane) of the metal components exposed to gas and traversed by the neutron beam is, therefore, of the order of 10 cm^2^.

**Figure 10:**
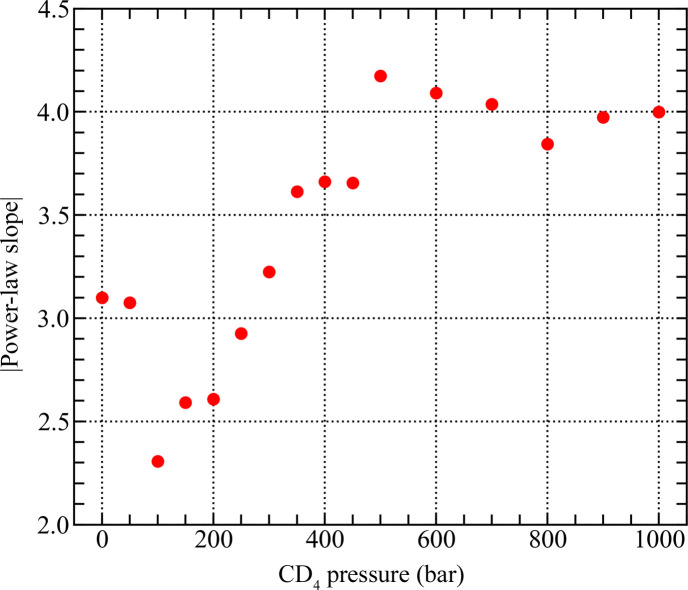
Evolution of the modulus of the power-law slope in the low-*Q* region

The surface area of the CD_4_/solid interface on the 100 nm scale can in principle be calculated from the average value of lim[*Q*^4^*I*(*Q*)] = 6.9 × 10^−9^ to 8.1 × 10^−9^ Å^−4^ cm^−1^, obtained from Porod plots at pressures higher than 500 bar (Fig. 10 ▸). The exact nature of the two scattering phases [and the scattering contrast to be used in equation (5)] in this region is uncertain, but the scattering intensity is almost unaffected by the CD_4_ pressure; therefore it is assumed that CD_4_ is condensed on the solid surface approximately to liquid CD_4_, with an SLD of ∼5.3 × 10^10^ cm^−2^. The SLD of the solid, meanwhile, can vary from −1.91 × 10^10^ cm^−2^ (for the titanium surface of the sample compartment) through 2.08 × 10^10^ cm^−2^ (for the aluminium body of the sample holder), 2.63 × 10^10^ cm^−2^ (for TiO_2_ of the oxidized titanium layer) and 2.19 × 10^10^ cm^−2^ (for SiO_2_ of the silica aerogel matrix) to 5.74 × 10^10^ cm^−2^ (for Al_2_O_3_ of the oxidized aluminium surface layer) (Table S3). The lower limit of the SSA, corresponding to scattering at the interface of Ti and CD_4_ (Table 1 ▸), is close to the macroscale (millimetre scale) surface area of the non-polished Al and Ti metal surfaces (∼10 cm^2^) exposed to CD_4_ and penetrated by the neutron beam inside the sample compartment. The low SSA obtained for the Ti–CD_4_ system is also consistent with the behaviour of the SANS results in this region: the intensity at CD_4_ pressures greater than or equal to 500 bar is 1.6 times the intensity from the silica aerogel in vacuum (Fig. 8 ▸) due to the higher contrast of the Ti–CD_4_ system. As a result, it is most likely that the low-*Q* scattering at CD_4_ pressures greater than or equal to 500 bar is dominated by the scattering of CD_4_ condensed on the surface of the titanium window.

**Figure 11:**
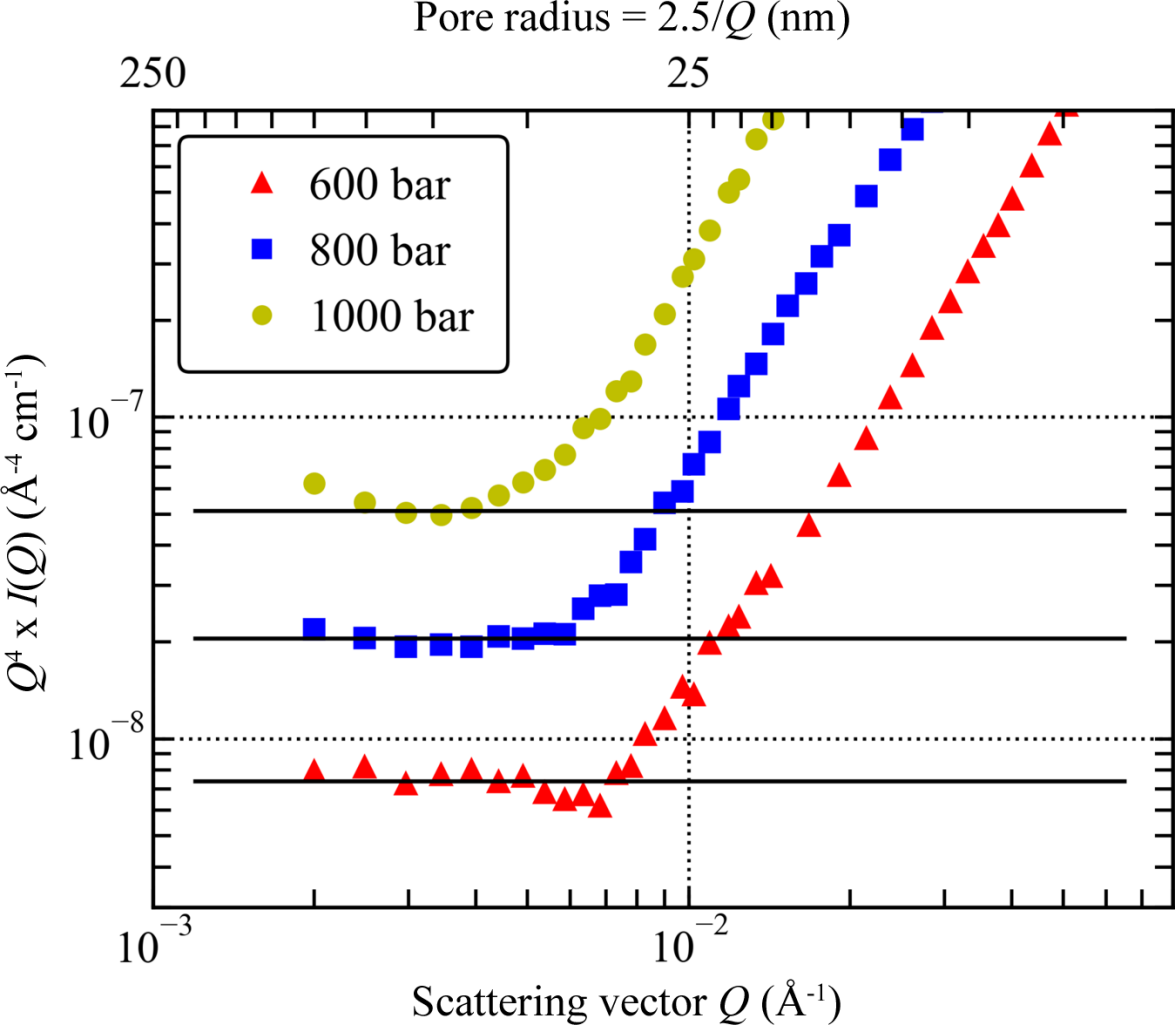
Porod plots of the silica aerogel at 600, 800 and 1000 bar of external CD_4_ pressure. Horizontal black lines represent the Porod limit fitted in the low-*Q* range. For clarity, the values of *Q*^4^*I*(*Q*) for the SANS data at 800 and 1000 bar are shifted up by factors of 2.5 and 7.5, respectively.

At CD_4_ pressures higher than or equal to 500 bar, the Porod limit is evident in all plots, indicating the formation of a smooth interface. However, at pressures lower than 800 bar the small-scale oscillations [equation (7)] are clearly seen in the Porod plots, in contrast to the *P* ≥ 800 bar data (Fig. 11 ▸). It is possible that the oscillations originate from a system of curved clusters of finite size, which evolve into a continuous phase at higher pressures of CD_4_. From the appearance of the Porod plot at 1000 bar, the peak of the Kirste–Porod correction [equation (6)], if it exists, is at a *Q* value outside the investigated *Q* range; therefore the size of CD_4_ clusters at high pressures can only be estimated as larger than 125 nm.

#### 4.2.5. Adsorption of CD_4_ in 2.5–50 nm pores

**Figure 12:**
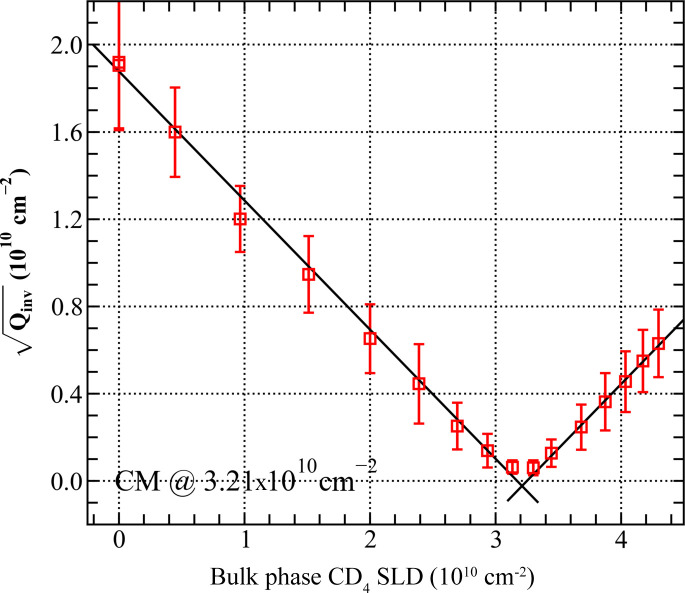
Square root of *Q*_inv_ as a function of bulk-phase CD_4_ SLD. Straight lines represent the scattering behaviour following the two-phase model.

For a two-phase system, the Porod invariant is proportional to the square of the scattering contrast 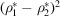 [equation (8)]; for this system composed of silica aerogel and pressurized CD_4_, it is expected that the contrast will be zero (at the CM point) at *P* = 415 bar, assuming that the density of CD_4_ in confinement is not different from the bulk density (Table S3). Fig. 12 ▸ shows a plot of (*Q*_inv_)^1/2^ versus SLD(CD_4_; *P*); *Q*_inv_ has been calculated over the entire *Q* range after subtraction of the high-*Q* and low-*Q* parasitic scattering from the measured SANS intensity, according to the procedure described in Appendix *C* in the supporting information. The plot is V-shaped and symmetric with respect to the reflection point at SLD = 3.21 × 10^10^ cm^−2^ [corresponding to *P*(CD_4_) of 415 bar and close to the SLD of amorphous silica of 3.46 × 10^10^ cm^−2^]. The remarkably low deviation of the two sections of (*Q*_inv_)^1/2^ from straight lines indicates a close-to-ideal two-phase interaction between the solid matrix of the silica aerogel and the pressurized CD_4_; it is concluded that the density of CD_4_ confined in the pores of the silica aerogel matrix in this *Q* range is close to the density of the bulk phase. The interaction with the silica aerogel matrix by supercritical CD_4_ differs from that reported for supercritical CO_2_, where the growth of a dense surface layer was observed, resulting in deviations from the two-phase approximation (Ciccariello *et al.*, 2011*a* ▸,*b* ▸).

The ratio of porosities calculated using equation (8), *Q*_inv_(CM)/*Q*_inv_(vac), is 0.001. This indicates that, as expected, the porous space of the silica aerogel is practically fully open to penetrating CD_4_, with an inaccessible porosity of 0.1%.

## 5. Conclusions

This study investigates the adsorption of d-methane (CD_4_) in silica aerogel pores (with a radius range of 0.6–125 nm) at a temperature of 22°C, using contrast-matched SANS (and partly USANS) in the pressure range from vacuum to 1000 bar. The highly porous structure of the aerogel (97% total porosity) has a mass-fractal-like distribution of pore sizes with a broad peak at *r* = 4 nm, which enables good insight into the scale-dependent adsorption process. We found several distinct sorption behaviours, which depend on the pore size.

(i) At the sub-nanometre and small-nanometre scale there is no evidence of CD_4_ condensation in the confinement; the SANS background is much higher than the expected in­coherent scattering of CD_4_.

(ii) In the pore radius range 5−50 nm the aerogel loaded with CD_4_ behaves like a classical two-phase system, with full contrast matching at *P* = 415 bar.

(iii) At the scales 50–125 nm (measured using SANS) and ∼5 µm (measured using USANS) there is evidence for the two-phase behaviour being progressively masked at increased pressures by a pressure-dependent parasitic scattering from the interfaces between the CD_4_ and the sample compartment components.

This study of a silica aerogel as a model system provides valuable supplementary information about the methane sorption mechanism in complex geological materials. The well defined structure and lack of contaminants in the aerogel facilitate a clear interpretation of the SANS results. The observed differences in CD_4_ uptake compared with geological materials may enable future optimization of methane storage strategies.

## 6. Related literature

For further literature related to the supporting information, see Chen *et al.* (1997 ▸) and Textor *et al.* (2001 ▸).

## Supplementary Material

Appendices A-D including supplementary figures and tables. DOI: 10.1107/S1600576724006794/ge5153sup1.pdf

## Figures and Tables

**Table 1:** Specific surface area (SSA) of the interfaces between different materials and liquid CD_4_, calculated using Porod fits to the SANS data in the low-*Q* region The CD_4_ SLD is 5.3 × 10^10^ cm^−2^ for liquid CD_4_. The SLD of SiO_2_ is 3.2 × 10^10^ cm^−2^. SLDs for metals and metal oxides are listed in Table S3 in the supporting information.

Bulk CD_4_ pressure (bar)	lim[*Q*^4^*I*(*Q*)] (10^−9^ Å^−4^ cm^−1^)	SSA Al (cm^2^ cm^−3^)	SSA Al_2_O_3_ (cm^2^ cm^−3^)	SSA Ti (cm^2^ cm^−3^)	SSA TiO_2_ (cm^2^ cm^−3^)	SSA SiO_2_ (cm^2^ cm^−3^)
500	6.91	106	5580	21.2	154	249
600	7.39	113	5970	22.6	165	267
700	7.81	120	6310	23.9	174	282
800	8.20	126	6620	25.1	183	296
900	8.13	125	6560	24.9	181	293
1000	6.81	104	5500	20.9	152	246
