# Supplementary material for: Revealing nanoscale sorption mechanisms of gases in a highly porous silica aerogel
Source: J Appl Crystallogr. 2024 Aug 19;57(Pt 5):1311–22. doi: 10.1107/S1600576724006794 (PMC11460400; doi:10.1107/S1600576724006794)
Supplement: Supplementary file 1 [file j-57-01311-sup1.pdf]

JOURNAL OF APPLIED CRYSTALLOGRAPHY

Volume 57 (2024)

Supporting information for article:

Revealing nanoscale sorption mechanisms of gases in highly porous silica aerogel

Phung Nhu Hao Vu, Andrzej P. Radlinski, Tomasz Blach, Ralf Schweins, Hartmut Lemmel, John Daniels and Klaus Regenauer-Lieb

Appendix A. SANS intensity of pressurized CD_4 at p=500 bar

The (unbound) incoherent scattering cross section of ^2H, the nucleus of deuterium ($2.0\cdot10^{-24}$ cm^2), is much smaller than its counterpart for hydrogen, ^1H, ($80.2\cdot10^{-24}$ cm^2); its contribution to the large-Q background scattering of rocks, with the typically accessible porosity of the order of several per cent, is of the order of 0.001 cm^{-1} at pressures about 1 kbar. This is markedly lower than the typical large-Q SANS background for geological materials (varying from ca. 0.01cm^{-1} for sandstones to ca. 1 cm^{-1} for organic-rich shale and coal) and can be safely neglected during the analysis of CM SANS results obtained using pressurized CD_4 (Zhang *et al.*, 2015; Bahadur *et al.*, 2018; Blach *et al.*, 2021; Sun *et al.*, 2022), compared to other contributions to the large-Q background (in particular, the molecular-scale compositional inhomogeneity and incoherent scattering on hydrogen nuclei bound in the organic matter, clays and formation fluids). The situation for the silica aerogel is different: it is chemically homogeneous and its porosity is exceptionally large (ca. 96%), hence the contribution of the ^2H incoherent scattering to the large-Q SANS intensity background may be significant even at the relatively low pressures of CD_4. Its magnitude at 500 bar is estimated to be $7\cdot10^{-3}$ cm^{-1}, since $(d\Sigma/d\Omega)_{incoh} = 1/4\pi\times\rho/M\times\sigma_i N_A F_A$, where $\sigma_i = 8.2\cdot10^{24}$ cm^2 is the incoherent cross section for ^2H bound in CD_4, $\rho = 0.345$ g/cm^3 is the bulk density of CD_4 at 500 bar, M is the molar mass of CD_4, N_A is Avogadro's number and F_A= 0.97 is the CD_4-accessible volume fraction inside the aluminium sample holder (accounting for the incomplete filling of the cell volume by the silica aerogel sample).

To verify this prediction, an additional control experiment – SANS measurement of the empty cell (without the Si-aerogel sample) – has been performed in vacuum and at the CD_4 pressure of 500 bar (above the contrast matching pressure of 415 bar). SANS intensity for the pressurized CD_4 fluid was calculated by comparing the two I(Q) curves of the empty cell at vacuum and under 500 bar of CD_4. The experimental SANS curves and the calculated scattering intensity of the bulk CD_4 fluid confined in the cell at p=500 bar (with and without the aluminium sample holder installed) are shown in Figure S1; the results indicate an unexpectedly large, Q-dependent scattering signal.

In particular, in the large-Q limit, the differential scattering cross sections measured at p=500 bar with and without the aluminium sample holder installed in the sample compartment are ~0.2 cm^{-1} and ~$5\cdot10^{-2}$ cm^{-1}, respectively, which is 25 and seven times larger than the theoretically predicted incoherent scattering value of $7\cdot10^{-3}$ cm^{-1}.

In the small-Q range (from 0.001 Å^{-1} to 0.01 Å^{-1}), the SANS intensity decreases from ~10^4 cm^{-1} to ~1 cm^{-1}, roughly according to the power law with the negative slope of -4.

The origin of the Q-dependent background scattering which so significantly exceeds the incoherent scattering of deuterium is not clear. Most likely it is caused by the heterogeneity of the scattering contrast on the sub-micrometre scale, due either to the density fluctuations of the supercritical CD_4 or scattering on the gas-metal interfaces inside the sample compartment, internal windows and the sample-window spacers exposed to the neutron beam. The latter hypothesis is supported by the fact that the intensity of the CD_4 scattering curves measured with and without the aluminium sample container in place are significantly different; it is examined in detail in the following sections.

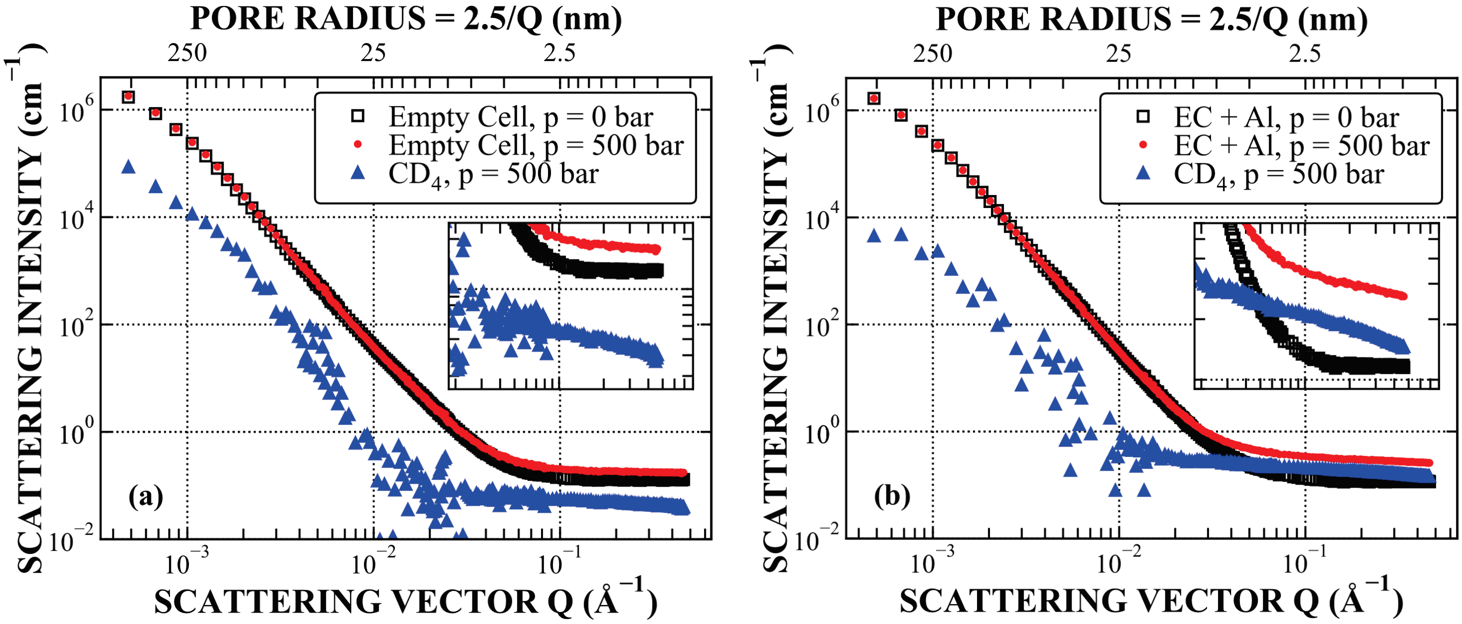

Figure S1 Comparison of SANS intensity measured for the evacuated empty cell and at 500 bar of CD_4 and the calculated scattering cross section of pressurized CD_4. The large-Q region detail is shown in the inset. (a) and (b) show SANS data for the empty cell with and without the aluminium sample holder installed in the sample compartment, respectively; the scattering intensities of CD_4 were calculated by subtracting the empty cell scattering measured in vacuum from that measured under the pressure of 500 bar, and are markedly different in magnitude.

Appendix B. Pressure dependence of SANS intensity in the large-Q region

In the large-Q range (from 0.1 to 0.5 Å^{-1}) the SANS intensity rapidly increases at the first pressure step from vacuum to 50 bar (Figure 6) and then remains relatively stable (at the level of about 0.1 cm^{-1}) with increased pressure; the SANS intensity tends to plateau rather than follow a V-shaped pressure dependence characteristic for a two-phase system subjected to contrast matching.

Importantly, the SANS patterns measured at p = 500 bar for the CD_4 fluid (acquired at ANSTO) and the CD_4-invaded silica aerogel sample (acquired at ILL) are similar (parallel and linear on the log-log plot),

with the slope value of -0.24. The intensities without the silica aerogel scale with a factor of 2.4 and 0.82 with and without the aluminium sample container installed, respectively, to the SANS data of the pressurized silica aerogel (Figure S2).

Following these observations, we postulate that in the large-Q region, the scattering intensity of silica aerogel invaded with pressurized CD_4 is dominated by the nano-scale heterogeneities of the scattering contrast which are not related to the presence of the aerogel sample hence they carry no useful silica aerogel structural information.

The scattering cross section measured at various pressures in the large-Q region is modelled using the power-law formula

$$I(Q)_{bkgrd} = \alpha Q^{\beta}, \tag{S1}$$

where α is the scattering intensity at Q = 1 $Å^{-1}$ and exponent β is the slope on the log-log plot. Parameters α and β fitted to Equation (S1) are shown in Figure S3 and listed in Table S1.

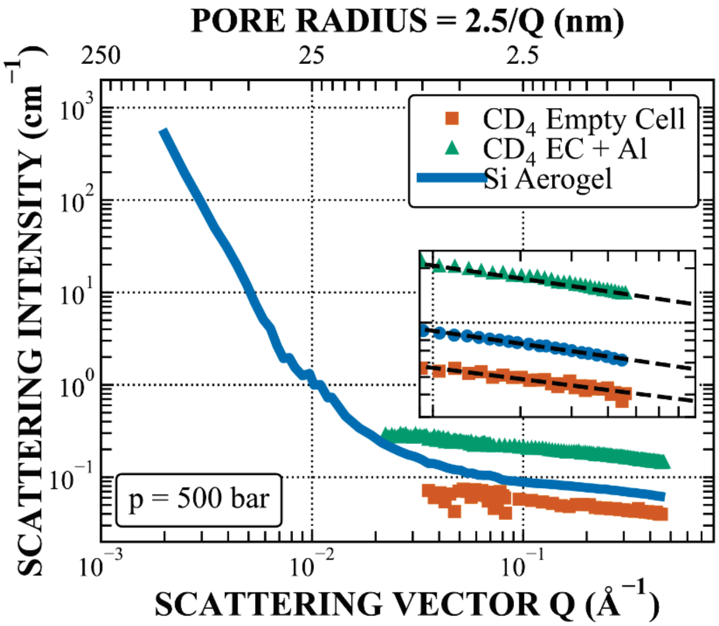

Figure S2 Comparison of SANS intensity measured at the CD_4 pressure of 500 bar for the empty cell, the empty cell with the aluminium container installed, and the silica aerogel sample mounted in the aluminium container. Inset shows details in the large-Q region; dashed lines represent power-law fits.

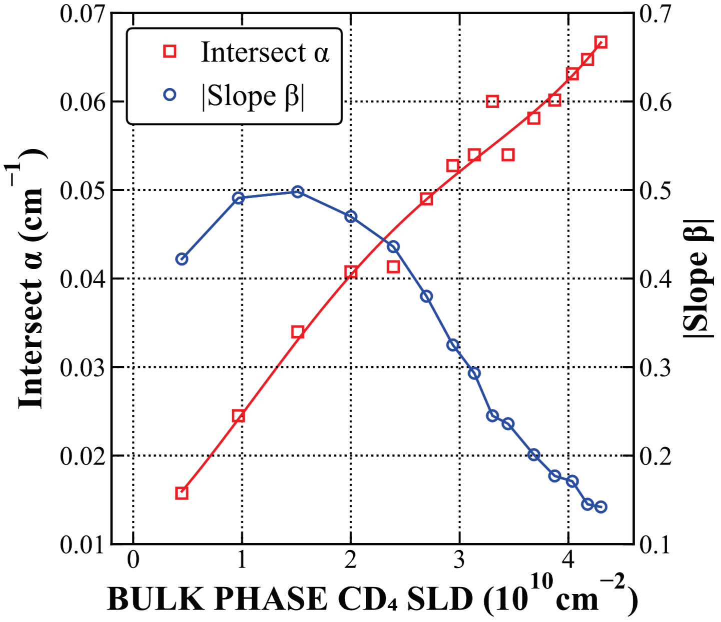

Figure S3 Parameters α and modulus of β obtained by fitting Equation (S1) to the pressure-dependent large-Q SANS data for the silica aerogel, as a function of SLD(CD_4)

Table S1 Parameters β (intersect with the vertical axis at Q = 1 Å^{-1}) and α (slope) obtained from fitting Equation (S1) to the large-Q SANS intensity for the silica aerogel.

Bulk CD_4 pressure	Intersect α (cm^{-1})	Slope β
50	0.015	-0.42
100	0.024	-0.49
150	0.033	-0.50
200	0.040	-0.47
250	0.040	-0.44
300	0.048	-0.38
350	0.051	-0.33
400	0.052	-0.29
450	0.058	-0.25
500	0.052	-0.24
600	0.056	-0.20
700	0.058	-0.18
800	0.061	-0.17
900	0.063	-0.15
1000	0.065	-0.14

Appendix C. Background scattering in the full Q-range

As discussed in previous sections and the main text, the scattering data at the large and low-Q limits, measured at $p(CD_4) > 0$, contains no structural information of the silica aerogel. We postulate that the background component (pressure-dependent and aerogel-independent) of the pressure-dependent SANS intensity can approximately be represented as a sum of two power-law curves fitted to the measured SANS intensity curves in the large-Q (Table S1) and small-Q (Table S2) end-regions and extrapolated to the mid-Q range; this is illustrated in Figure S4(a) for SANS data acquired at p=150 bar and in Figure S4(b) as a colour map for the entire pressure range. To measure the variation of the scattering contrast with pressure in the mid-Q region we use the Porod Invariant [Equation (8)] rather than the square root of I(Q) at a fixed Q-value, which has the advantage of (i) weighing the result with Q^2, hence partly compensating for the approximate character of the Q^{-4} representation of the small-Q background component, especially at the low pressures and (ii) providing a trend for the average over a wider Q range, rather than isolated to a particular Q value.

We note that using the power law fitted to the low-Q scattering at CD_4 pressure lower than 200 bar is an overestimation of the background, e.g. the pressure of 150 bar shown in Figure S4(a). At $p(CD_4) <$ 200 bar, the scattering at the low-Q regions most likely consists of the scattering from (i) the large-scale rough-surface fractal structure of the silica aerogel and the supercritical CD_4, and (ii) the smooth surface scattering from the CD_4-metal surface. This Q^2-weighted inaccuracy is inconsequential for the calculations of Invariant, as demonstrated in Figure S5 which compares the pressure dependence of Porod Invariant calculated after the subtraction of (i) sum of the small-Q power-law (350-1000 bar) and large-Q power-law (50-1000 bar) and (ii) sum of the small-Q power-law (50-1000 bar) and the large-Q power-law (50-1000 bar).

Table S2 Parameters β (intersect with the vertical axis at Q = 1 Å^{-1}) and α (slope) obtained from fitting Equation (S1) to the low-Q SANS intensity for the silica aerogel.

Bulk CD_4 pressure	Intersect α (cm^{-1})	Slope β
50	-5.83	-3.08
100	-3.96	-2.31
150	-4.82	-2.59
200	-4.99	-2.61
250	-5.80	-2.93
300	-6.53	-3.22
350	-7.45	-3.61
400	-7.45	-3.66
450	-7.41	-3.66
500	-8.57	-4.17
600	-8.35	-4.09
700	-8.20	-4.04
800	-7.70	-3.84
900	-8.02	-3.97
1000	-8.13	-4.00

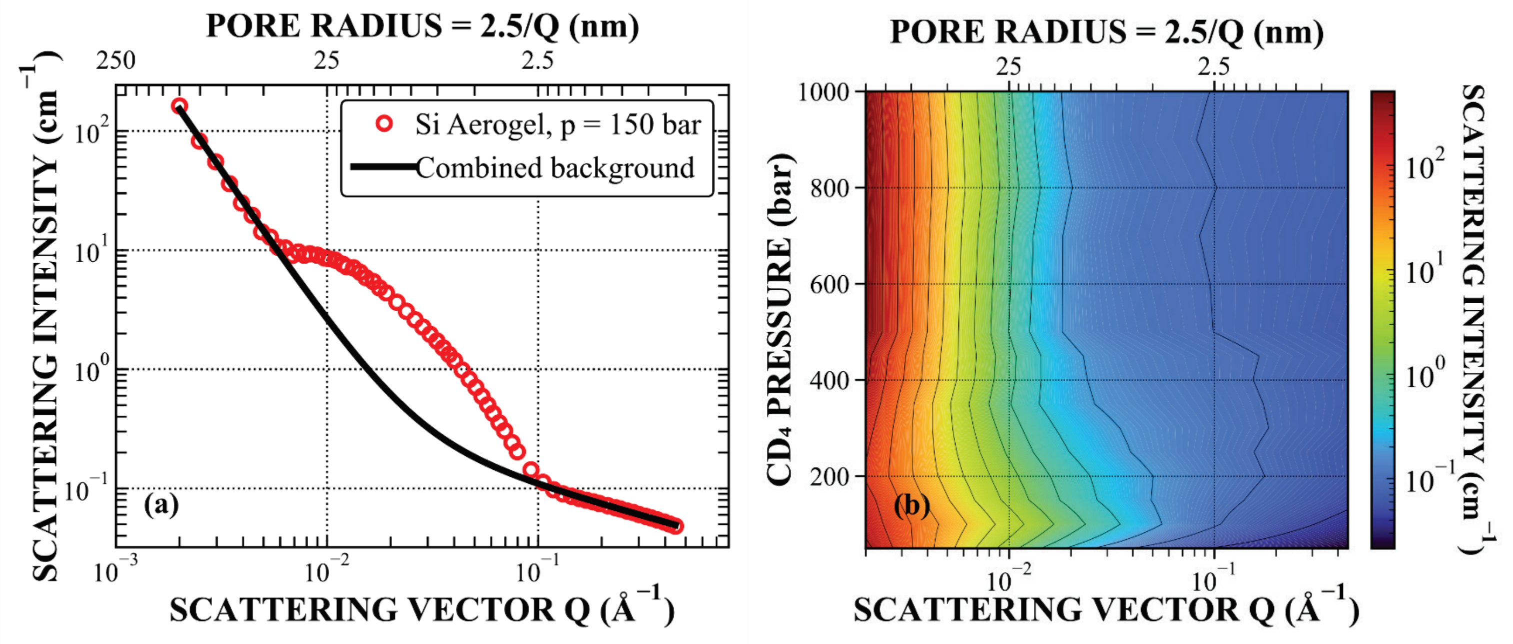

Figure S4 Q dependence of the silica-aerogel-independent background at the CD_4 pressure of 150 bar; it is the sum of extrapolated power-law scattering intensities fitted in the low-Q and large-Q regions: (a) calculated for SANS results acquired at p(CD_4) = 150 bar; (b) colour map of the background for various pressures of CD_4.

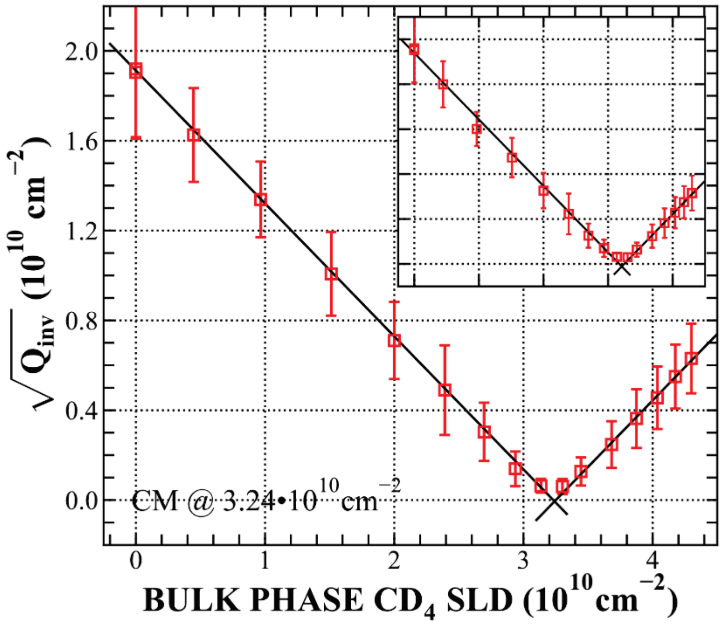

Figure S5 Pressure dependence of the square root of the Porod Invariant calculated after the subtraction of two different backgrounds from the original SANS results. Main panel: small-Q power-law (350-1000 bar) plus large-Q power-law (50-1000 bar); Inset: small-Q power-law (50-1000 bar) plus large-Q power-law (50-1000 bar), shown previously in Figure 11.

Appendix D. Contrast matching of pressurized CD_4 with the sample compartment components

This section summarizes the scattering length densities of solids which are exposed to CD_4 inside the sample compartment volume transected by the neutron beam, and compares them with the scattering length density of CD_4 at various pressures.

Table S3 SLD values for the sample compartment metal components exposed to pressurized CD_4. Ranges of thicknesses of the naturally formed surface layers of oxides are the typical values, reported after exposure to air at room temperature for times from days to a year (Chen *et al.*, 1997; Textor *et al.*, 2001). Values of SLD were calculated using the NIST calculator (NIST Center for Neutron Research).

Metal component chemical formula	Density (g/cm^3)	SLD (10^{10} cm^{-2})	pCD_4 at CM point (bar)	Typical layer thickness (nm)
Al	2.70	2.078	209	bulk
Al_2O_3	4.0	5.744	> 2 kbar	2 - 7
Ti	4.506	-1.910	CM not possible	bulk
TiO_2	4.23	2.628	288	2 - 6
SiO_2 (silica aerogel matrix)	2.05	3.19	415	bulk
CD_4 at p=1 kbar at 22ºC	0.43	4.3	n/a	bulk

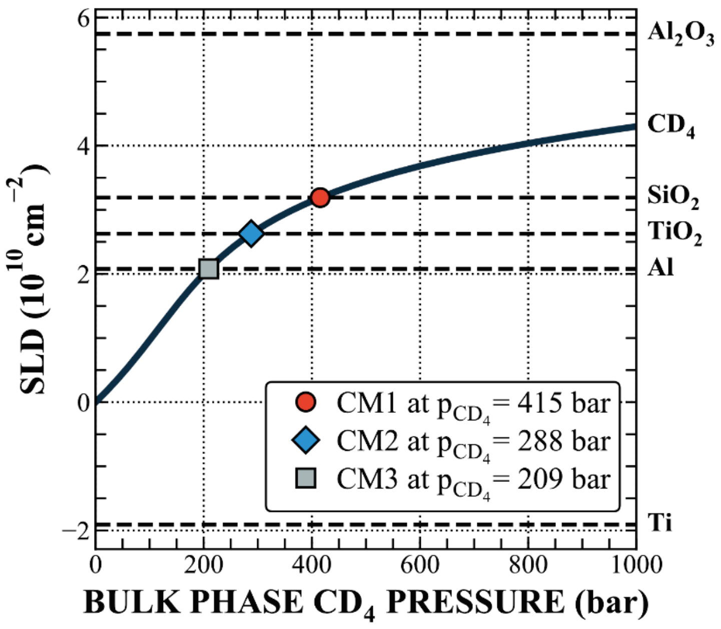

Figure S6 Diagram showing the pressures of CD_4 and corresponding SLD values at the contrast match (CM) points (where appropriate) with titanium, titanium oxide, aluminium, aluminium oxide and the silica aerogel solid matrix. Data for CD_4 were taken from the NIST Chemistry WebBook (NIST Chemistry WebBook); SLDs for solids are listed in Table S3. The CM pressures are 415 bar for the silica aerogel matrix (SLD=$3.2\cdot10^{10}$ cm^{-2}, marked CM1), 288 bar for TiO_2 (SLD=$2.6\cdot10^{10}$ cm^{-2}, marked CM2) and 209 bar for Al (SLD=$2.1\cdot10^{10}$ cm^{-2}, marked CM3 in the plot). The CM pressure for Al_2O_3 (>2 kbar) is outside of the experimental range; titanium has a negative SLD which cannot be matched using CD_4. CM conditions close to CM1 are observed in the mid-Q pore size range of 2.5 nm to 25 nm (Figure 12; nearly full contrast match) and those close to the combined CM2 and CM3 are seen for the ~125 nm pore sizes (Figure 8, the largest pore size investigated; about 70% contrast match)

References

Bahadur, J., Ruppert, L. F., Pipich, V., Sakurovs, R. & Melnichenko, Y. B. (2018). *International Journal of Coal Geology* **188**, 156-164.

Blach, T., Radlinski, A. P., Vu, P., Ji, Y., de Campo, L., Gilbert, E. P., Regenauer-Lieb, K. & Mastalerz, M. (2021). *Energies* **14**, 8438.

Chen, C., Splinter, S. J., Do, T. & McIntyre, N. S. (1997). *Surface Science* **382**, L652-L657.

NIST Center for Neutron Research *Neutron activation and scattering calculator,* https://www.ncnr.nist.gov/resources/activation/.

NIST Chemistry WebBook *Isothermal Properties for Methane,* https://webbook.nist.gov/cgi/fluid.cgi?Action=Load&Applet=on&ID=C74828&Type=IsoTherm&Digits=5&PLow=0&PHigh=1000&PInc=50&T=22&RefState=DEF&TUnit=C&PUnit=bar&DUnit=g%2Fml&HUnit=kJ%2Fmol&WUnit=m%2Fs&VisUnit=uPa*s&STUnit=N%2Fm.

Sun, M., Wen, J., Pan, Z., Liu, B., Blach, T. P., Ji, Y., Hu, Q., Yu, B., Wu, C. & Ke, Y. (2022). *International Journal of Coal Geology* **255**, 103987.

Textor, M., Sittig, C., Frauchiger, V., Tosatti, S. & Brunette, D. M. (2001). *Titanium in Medicine: Material Science, Surface Science, Engineering, Biological Responses and Medical Applications*, edited by D. M. Brunette, P. Tengvall, M. Textor & P. Thomsen, pp. 171-230. Berlin, Heidelberg: Springer Berlin Heidelberg.

Zhang, R., Liu, S., Bahadur, J., Elsworth, D., Melnichenko, Y., He, L. & Wang, Y. (2015). *Fuel* **161**, 323-332.
